# Aberrant Promoter Methylation and Expression of UTF1 during Cervical Carcinogenesis

**DOI:** 10.1371/journal.pone.0042704

**Published:** 2012-08-03

**Authors:** Samuel Guenin, Mustapha Mouallif, Rachel Deplus, Xavier Lampe, Nathalie Krusy, Emilie Calonne, Katty Delbecque, Frederic Kridelka, François Fuks, My Mustapha Ennaji, Philippe Delvenne

**Affiliations:** 1 Laboratory of Experimental Pathology, Groupe Interdisciplinaire de Génoprotéomique Appliquée (GIGA)-Cancer, University of Liège, Liège, Belgium; 2 Laboratory of Virology and Hygiene and Microbiology, University Hassan II-Mohammedia, Mohammedia, Morocco; 3 Laboratory of Cancer Epigenetics, Free University of Brussels, Brussels, Belgium; 4 Department of Pathology, CHU Sart Tilman, Liège, Belgium; 5 Department of Obstetrics and Gynecology, CHU Sart Tilman, Liège, Belgium; University of Navarra, Spain

## Abstract

Promoter methylation profiles are proposed as potential prognosis and/or diagnosis biomarkers in cervical cancer. Up to now, little is known about the promoter methylation profile and expression pattern of stem cell (SC) markers during tumor development. In this study, we were interested to identify SC genes methylation profiles during cervical carcinogenesis. A genome-wide promoter methylation screening revealed a strong hypermethylation of Undifferentiated cell Transcription Factor 1 (UTF1) promoter in cervical cancer in comparison with normal ectocervix. By direct bisulfite pyrosequencing of DNA isolated from liquid-based cytological samples, we showed that *UTF1* promoter methylation increases with lesion severity, the highest level of methylation being found in carcinoma. This hypermethylation was associated with increased *UTF1* mRNA and protein expression. By using quantitative RT-PCR and Western Blot, we showed that both UTF1 mRNA and protein are present in epithelial cancer cell lines, even in the absence of its two main described regulators Oct4A and Sox2. Moreover, by immunofluorescence, we confirmed the nuclear localisation of UTF1 in cell lines. Surprisingly, direct bisulfite pyrosequencing revealed that the inhibition of DNA methyltransferase by 5-aza-2′-deoxycytidine was associated with decreased *UTF1* gene methylation and expression in two cervical cancer cell lines of the four tested. These findings strongly suggest that *UTF1* promoter methylation profile might be a useful biomarker for cervical cancer diagnosis and raise the questions of its role during epithelial carcinogenesis and of the mechanisms regulating its expression.

## Introduction

Cervical cancer is the third cause of cancer-related death in women worldwide after breast and colon cancer [1]. Persistent infection with an oncogenic type of human papillomavirus (HPV) is a necessary factor for the development of invasive squamous cell carcinoma (SCC) of the cervix [2]. SCC are preceded by squamous intraepithelial lesions (SIL). Although the majority of low grade SIL (LSIL, corresponding to CIN1 and condyloma) spontaneously regress, high grade SIL (HSIL, corresponding to CIN2 and CIN3), if left untreated, can progress to SCC [3].

One of the hallmarks of carcinogenesis is the specific hypermethylation of CpG islands within the promoter of tumor suppressor genes, which usually results in the silencing of these genes leading to anarchical cell growth, proliferation and ultimately to the formation of invasive tumor and metastasis [4], [5]. It has also been established that embryonic development and cancer pathogenesis share several molecular features. Accordingly, regulation of embryonic stem cell (SC) markers became of particular interest since it has been proposed that they are expressed by a small subpopulation of cells within tumor mass. The so-called tumor initiating cells or cancer stem cells (CSC) drive all the invasive properties of the tumor [6], [7]. The presence of CSC has been recently suggested in cervical cancers [8], [9].

However, the expression pattern and epigenetic regulation of SC markers in human epithelial tumors, as well as their clinical significance, are still unclear. Recent investigations about *OCT4* gene have shown that, despite its promoter hypermethylation in cervical cancer cell lines, this gene is expressed whereas another study indicated the absence of Oct4A, the specific isoform of SC, in epithelial cancer cell lines [10], [11]. Moreover, confusing data on *OCT4* and *NANOG* could be related to the existence of multiple isoforms and pseudogenes (at least 6 and 10 pseudogenes for *OCT4* and *NANOG*, respectively) [12], [13]. In prostate tumors, *CD133* expression, another CSC marker, was not correlated to its promoter methylation status, whereas in colon cancer, the CD133 negative cells were shown to form tumors much more aggressive than their CD133 positive counterparts [14], [15]. In cervical cancer, SC markers *SOX2* and *SMOOTHENED* were recently shown to be overexpressed by nearly all the cells within tumour mass [16], [17].

Another specific agent regulating SC functions is the Undifferentiated cell Transcription Factor 1 (*UTF1*). Human *UTF1* gene codes for only one isoform, and up to now, no pseudogene has been found [18]. Its expression was described to be restricted to SC, teratocarcinoma cell lines and testicular germ cell tumors [18]–[21]. Recent studies indicated that UTF1 is a stably chromatin-associated transcriptional repressor protein involved in the initiation of SC differentiation, but not in the SC self-renewal [19], [20], [22]. During development, in adult somatic tissues, its expression is downregulated and hypermethylation of the Oct4/Sox2 enhancer, upstream the coding region of the gene, was proposed as a mechanism participating to its inhibition [22]. In a cancer context, UTF1 was shown to be highly expressed in germ cells tumors [19], [21]. However, in somatic tumors, UTF1 expression was suggested to be of potential interest to differentiate between grade I–III and grade IV neuroblastoma tumors whereas it was shown to be expressed at the same level in healthy epidermis and in skin SCC [23], [24].

In this study, we were interested to identify SC gene methylation signatures during cervical carcinogenesis. We demonstrated that *UTF1* gene promoter is hypermethylated and associated with UTF1 overexpression in cervical SCC in comparison with normal ectocervix. *In vitro* experiments allowed to detect UTF1 in the nucleus of epithelial somatic cancer cell lines even in the absence of its main known regulators, Sox2 and Oct4A. Our results suggest that *UTF1* promoter methylation profile could be used as a biomarker for cervical cancer diagnosis and raise the questions about the mechanisms involved in the regulation of its expression in epithelial tumors.

## Materials and Methods

### Ethics statements

The study involving human biopsy samples was conducted in accordance with the Declaration of Helsinki and approved by the local ethics committee of the University Hospital of Liege (reference: B70720109045). Patients gave written informed consent for the sample collection.

### Tissue collection

For frozen tissues, 4 samples of Ecto originating from total hysterectomy for non cervical pathologies and 5 samples of SCC were used. For Tissue microarray (TMA) construction, 66 paraffin-embedded tissue blocks of conisation or hysterectomy specimens representing 48 cases including various (pre)neoplastic cervical squamous intraepithelial lesions (11 LSIL, 18 HSIL and 19 SCC) and 18 tumor-free ectocervix samples (Ecto) from hysterectomy for noncervical pathologies were collected [25]. Liquid-based cytology samples (LBC) were composed of 17 Ecto, 10 LSIL, 11 HSIL and 9 SCC.

### Laser Capture Microdissection and DNA isolation

Serial frozen sections (10 µm thick) of 5 SCC and 4 Ecto were obtained using a Microm HM 500 M cryostat (Microm International, Francheville, France) and mounted on glass slides covered with a thin membrane (Carl Zeiss Microscopy, Munich, Germany). Sections were then stained with Gill III hematoxylin (RNase free, Merck, Darmstadt, Germany) for 1 minute, washed in distilled water and in ethanol, and dried at RT. Microdissection was performed using a P.A.L.M. microdissector (Carl Zeiss Microscopy) and was supervised by a histopathologist to ensure >95% specificity of captured cells.

### DNA isolation and bisulfite conversion

DNA from microdissected tissue was extracted using QIAamp DNA micro kit (Qiagen, Valencia, CA) according to manufacturer's instructions. DNA from LBC samples was extracted using Nucleospin® Tissue kit (Macherey-Nagel, Düren, Germany) according to manufacturer's instruction. DNA concentration and quality was assessed with a ND-1000 spectrophotometer (NanoDrop, Wilmington, DE). Bisulfite conversion of DNA was performed with EZ DNA Methylation kit (Zymo Research, Irvine, CA) for methylation microarray (500 ng DNA/sample) and methyl-specific PCR (1 µg DNA/sample) or with Epitect bisulfite kit (Qiagen) for BP analyses (250 ng DNA/sample) according to manufacturer's instructions.

### Methylation microarray and bioinformatic analysis

Microarrays were performed by the GIGA-Genotranscriptomic platform (GIGA, Liège University, Belgium). The HumanMethylation27 BeadChip (Illumina, San Diego, CA) has been described previously [26]. After bisulfite conversion, DNA was whole-genome amplified, enzymatically fragmented and 200 ng of DNA was used for microarray. After extension, the array was fluorescently stained, scanned and the intensities of the non-methylated and methylated bead types were measured. DNA methylation values, described as β-values, were recorded for each locus of each sample and processed with BeadStudio (Illumina). All the beads of one probeID from all samples in each group (Ecto and SCC) were aggregated to calculate the mean values, standard deviations, detection p-values and β-values of the given probeID. The detection *p*-value is defined as the minimum of the 2 separate p-values of the 2 variables (green A/B or red A/B), where each *p*-value is the result of testing (Mann-Whitney-U-test) against the negative beads of each channel. The Benjamini-Hochberg correction was applied before choosing the minimum p-value [27]. To compare methylation between Ecto and SCC, the difference of β-values of the two groups was tested using Mann-Whitney-U-test. Clustering was performed using Cluster 3.0 [28]. The MIAME-compliant microarray data are available in GEO (GEO accession number GSE36637).

### Direct bisulfite pyrosequencing

Bisulfite converted DNA was eluted in 20 µL of elution buffer and was subjected to PCR amplification of the specific region by use of a primer set designed to amplify both methylated and unmethylated sequences of the *UTF1* gene promoter. The primers were designed using PSQ assay design software (Qiagen). One of the primers was biotin labelled. The primers sequences (in 5′- -3′ orientation) are, for the first step, forward: AGGGGTTTTAGTTTTTTTAGTAGAGGTGTT-Btn and reverse: AACCCCTAACCCAATAACAAACT and for sequencing: GGGGGAGGATGTTAAG. Hot start Taq DNA polymerase High fidelity (Qiagen) was used to perform the PCR reaction. The PCR product was checked by 1.5 % agarose gel electrophoresis to confirm the quality, the size of the product and rule out the formation of primer dimers. The specific PCR products were then subjected to quantitative pyrosequencing analysis using a Biotage PyroMark Q24 system (Qiagen) according to manufacturer's instruction. The results were analyzed by Pyro Q-CpG 1.0.9 software (Qiagen). Based on control normal samples and internal quality controls provided in the software analysis, a cut-off value was set at 20%, meaning that CpG methylation above this limit was considered as hypermethylated, whereas below 20% was considered as hypomethylated [27], [29].

### White Blood Cell isolation

Human white blood mononuclear cells (WBC) were obtained as previously described [30]. Briefly, WBC were isolated from leukocyte-enriched buffy coats by centrifugation on Ficoll-Hypaque. After washings at low centrifugation speed to discard a maximum of platelets, WBC were pelleted, washed with PBS and stored at −80°C until DNA extraction.

### RNA isolation and PCR analyses

One µg of total RNA extracted from cell cultures or LBC samples (NucleoSpin RNAII, Macherey-Nagel), treated with DNase and quantified with a ND-1000 spectrophotometer was reverse transcribed using Superscript II reverse transcriptase (Invitrogen, Gent, Belgium) according to the manufacturer's instructions. The cDNA were stored at −20°C until use. Quantitative Real Time PCR was performed for *UTF1* using Taqman Gene Expression Assay (Applied Biosystems, Carlsbad, CA) primers (Hs00864535_s1) and normalized against *GAPDH* (Hs99999905_m1). Each sample was analyzed in triplicate. For UTF1 RT-PCR reactions, a set of previously described primers was used: Forward, 5′-CGCCGCTACAAGTTCCTTAAA-3′ ; Reverse, 5′-GGATCTGCTCGTCGAAGGG-3′ (annealing temperature 55°C, 35 cycles, 76 bp) (Figure S1A) [21]. Housekeeping gene (GAPDH) primers were: Forward, 5′-TGATGACATCAAGAAGGTGGTGAAG-3′; Reverse, 5′-TCCTTGGAGGCCATGTGGGCCAT-3′ (annealing temperature 60°C, 35 cycles, 240 bp). Samples were run on 1.8% agarose gels containing ethidium bromide and visualized with an UV transilluminator.

### Tissue microarray construction

TMA was constructed as previously described [25]. Briefly, the tissue areas (validated by a histopathologist) used for the construction of TMA were selected on the haematoxylin/eosin slides and on the donor blocks and were sampled using a manual arraying instrument (Beecher Instruments, Sun Prairie, WI). TMA blocks were constructed using 1-mm tissue cores (Alphelys, Plaisir, France). Sections of 5 µm were performed and were coated with paraffin for future use.

### Immunohistochemistry

Sections were deparaffinized, rehydrated in graded alcohols and antigens were retrieved in citrate buffer by microwave heating (5 min 750 W, 15 min 300 W). After endogenous peroxydase inhibition, nonspecific binding sites were blocked with Protein Block Serum-Free solution (Dako, Glostrup, Denmark). Slides were incubated overnight at 4°C with a mouse monoclonal antibody against UTF1 (dilution 1/250, clone 5G10.2, Millipore, Temecula, CA). The specificity of the anti-UTF1 antibody was checked by Western Blot in cells overexpressing the human protein (Figure S1B). Immunoperoxidase staining was performed using the LSAB2 kit (Dako). A negative control was obtained by omitting the primary antibody. Spermatogonia in human testicles were chosen as positive control, as previously reported [21]. Positive cells were visualized using a 3,3′-diaminobenzidine (DAB) substrate, and the sections were counterstained with Meyer haematoxylin. A score for the staining was established by determining the intensity and extent of the staining according to an arbitrary scale. For staining intensity, 0 represented samples in which the immunoreactivity was undetectable, whereas 1, 2, and 3 denoted samples with, respectively, a low, moderate, and strong staining. For staining extent, 0, 1, 2, and 3 represented samples in which the immunoreactivity was detectable, respectively, in <5%, 6% to 25%, 26% to 75% and >75% of the tumor cells. To provide a global score for each case, the results obtained with the two scales were multiplied, yielding a single scale of 0, +1, +2, +3, +4, +6 and +9 [30].

### Cell Culture

DMEM (Gibco, Carlsbad, CA) and RPMI (Gibco) were supplemented with 10% foetal bovine serum (Gibco), 2 mM L-glutamine (Gibco), 1% Sodium pyruvate (Gibco), 1% non essential amino acids (Gibco), 100 U/ml penicillin G, 100 µg/ml streptomycin (Gibco). Cell cultures were passaged every three days The cancer cell lines derived from the anogenital area A431, CaSki, SiHa, and Hela (all from the American Type Culture Collections (ATCC)) were grown in DMEM. The teratocarcinoma NCCIT cell line (from the ATCC, kind gift of Pr Luc Grobet, Laboratory of Animal Histology and Embryology, Faculty of Veterinary Medicine, University of Liege, Belgium) treated or not with retinoic acid (all-trans retinoic acid, 10 µM, 8 days, Sigma, St Louis, MO) in RPMI was used as negative and positive controls for *UTF1* expression, respectively [18]. For DNA-methyltransferase inhibition, cells were treated with 5-aza-2′-deoxycytidine (Sigma) at 10 µM for 72 hrs, with medium changed every day. Then cells were pelleted and stored at −80°C until analysis.

### Protein extraction and western blot analysis

Cells were lysed in RIPA buffer (Pierce, Rockford, IL) supplemented with 1 mmol/L phenylmethyl sulfonyl fluoride (Sigma), and protease inhibitors (Roche). After quantification (BCA protein assay, Pierce), proteins were separated by electrophoresis on 4% to 12% NuPAGE polyacrylamide gels (Invitrogen) and transferred onto polyvinylidene difluoride membranes. The membranes were subsequently blocked with 5% skim milk for 30 minutes and incubated overnight at 4°C with anti–β-actin (Sigma), anti-UTF1 (mouse monoclonal clone 5G10.2, Millipore), anti-Oct4A (mouse monoclonal sc-5279, Santa Cruz Biotechnology, CA [31]) or anti-Sox2 (rabbit monoclonal clone D6D9, Cell Signalling, Danvers, MA). The membranes were then incubated with appropriate secondary antibodies (Amersham Biosciences, Piscataway, NJ). After washing, proteins were detected using an enhanced chemiluminescence system (ECL Plus, Amersham Biosciences).

### Effect of 5-aza-2′-deoxycytidine on cell proliferation and viability

For the proliferation assay, cells were seeded in 6-well plates and allowed to attach for 24 hrs. Then the cells were treated or not with 5-aza for 72 hrs as described in the section “Cell Culture”. Every 24 hrs, adherent cells were harvested and counted. Experiments were performed twice in triplicate. Cell viability was evaluated using a classical MTT reduction assay (Roche, Vilvoorde, Belgium) according to the manufacturer's instructions [32].

### Immunofluorescence

Immunofluorescence was performed as previously reported [18]. Cells were plated in two well Lab-Teck slides for 48 h in DMEM. After washing with 0.5 M Phosphate Buffer Saline 1X (PBS), cells were fixed with 4% paraformaldehyd for 15 minutes at room temperature, and then permeabilized with 0.5% of Triton-X100 for 5 min followed by washings in PBS. Slides were incubated overnight at 4°C with anti-UTF1 antibody (1/600, clone 5G10.2, Millipore) or antibody diluents without anti-UTF1 antibody as negative control. Goat-anti mouse labeled with rhodamine (1/500, Invitrogen) was used as secondary antibody. Before mounting, slides were briefly washed with dH2O and cell nuclei stained with 4′,6-diamino-2-phenylindole (DAPI, 1/2000, Boehringer Ingelheim, Ingelheim am Rhein, Germany) for 5 min. After mounting slides with Eukitt, pictures were acquired using an epifluorescence microscope (Carl Zeiss Inc., Oberkochen, Germany).

### Data analysis

All results are given as mean values ± SE. Statistical analyses and graphical representations were performed with GraphPad Prism 5 software (San Diego, CA). All results were considered significant when *p*-values were less than 0.05. Statistical tests used are indicated in Figure legends.

## Results

### Methylation status of stem cell genes in normal and cancer tissues of the uterine cervix

**Figure 1:**
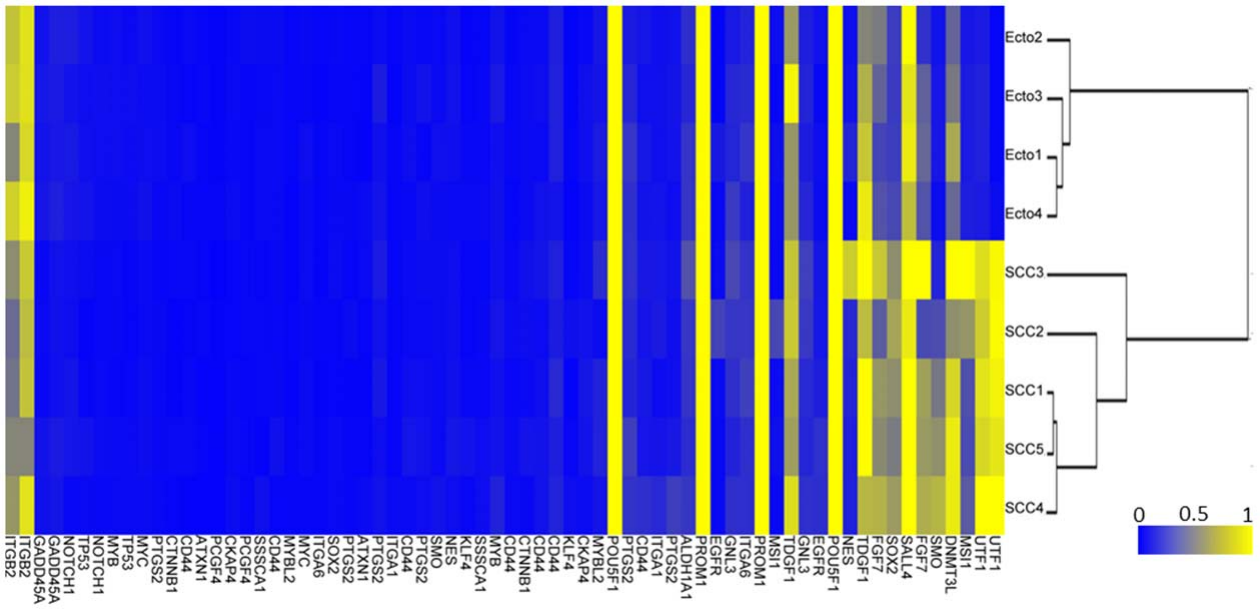
Hierarchical cluster analysis of methylation levels of genes involved in stem cell physiology. Gene methylation was analyzed by Illumina microarray in DNA from microdissected normal ectocervix (Ecto1 to 4) and cervical squamous cell carcinoma (SCC1 to 5).

We first analyzed by microarray the methylation profile of 31 genes (68 CpG) specific for SC in the DNA isolated from 4 normal Ecto and 5 cervical SCC. A mean β-value for each CpG was calculated for the 5 SCC and compared with the mean β-value for the 4 Ecto (Δβ). We then applied a threshold with a Δβ>0.2 and *p*<0.05 (Mann-Whitney U-test) in order to identify hypermethylated genes in SCC [27], [29]. Values are given in Table S1. Using an unsupervised hierarchical cluster analysis we obtained two groups, one encompassing the 4 Ecto and the other group the 5 SCC (Figure 1). Only 7 genes exhibited a Δβ>0.2 with *p*<0.05 (Figure 1): *UTF1* (2/2 CpG), *FGF7* (1/2 CpG), *MSI1* (1/2 CpG), *SOX2* (1/2 CpG), *SMO* (1/2 CpG), *DNMT3L* (1/1 CpG) and *SALL4* (1/1 CpG). We then focused our experimentations on *UTF1*, since the two CpG of this gene were the most hypermethylated in SCC (Δβ = 0.685 and 0.545, *p*<0.05 for each CpG). This gene is composed of two exons separated by a short intron. The Oct4/Sox2 enhancer is located upstream the 3′ sequence, (Figure S2). The two CpG analyzed in our microarray are located in a CpG island covering the promoter region, the transcription start signal and the first exon (red triangles, Figure S2).

### Methylation status of UTF1 promoter in DNA isolated from liquid-based cytological samples

**Figure 2:**
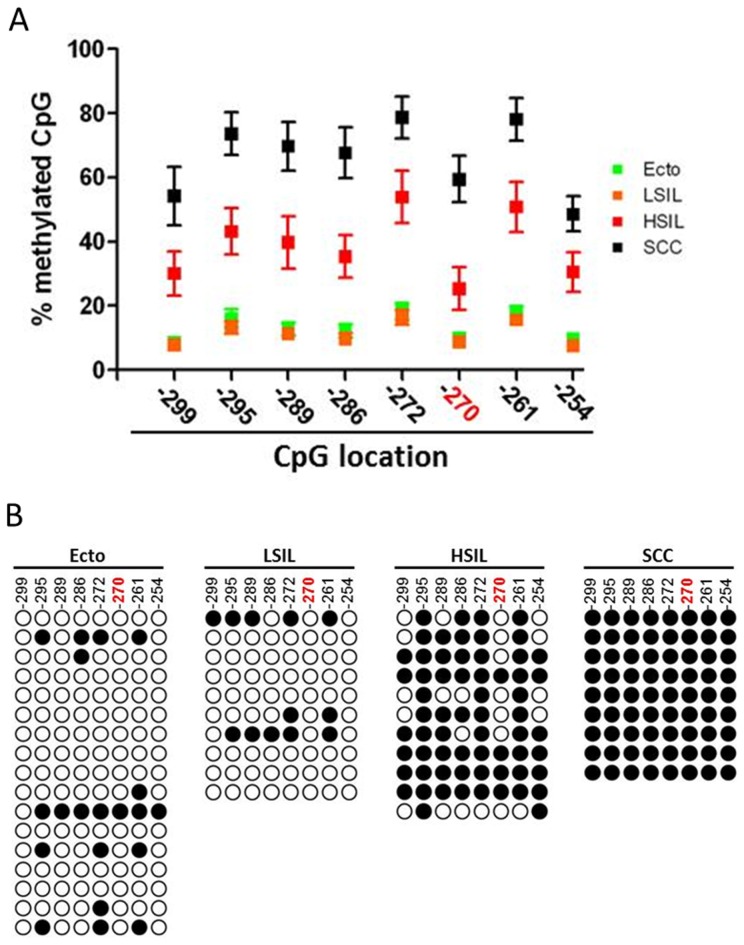
*UTF1* promoter methylation increases during cervical carcinogenesis. A) Mean methylation value for each CpG analysed by direct bisulfite pyrosequencing. Position of each CpG is indicated (TSS = +1). B) CpG methylation profile in each sample (cut-off value of 20%). Each line represents one sample. Ecto, normal ectocervix; LSIL, low grade squamous intraepithelial lesion; HSIL, high grade squamous intraepithelial lesion; SCC, squamous cell carcinoma; white circle, hypomethylated CpG; black circle, hypermethylated CpG. The CpG in red was the one analyzed by microarray.

**Figure 3:**
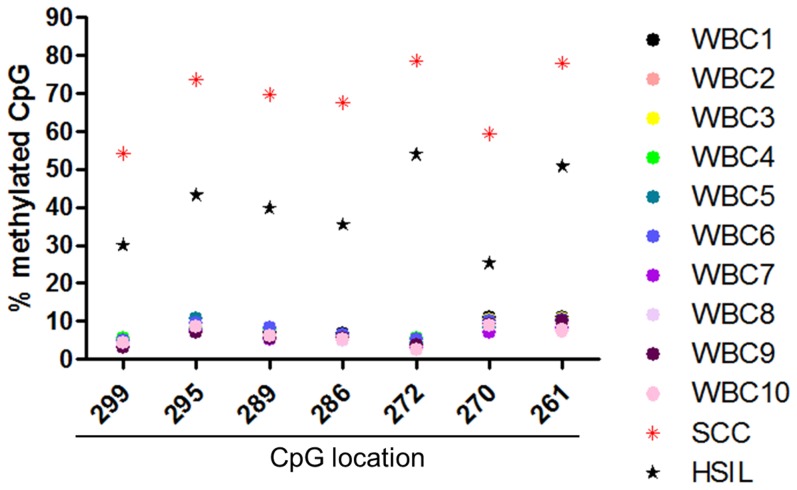
*UTF1* promoter methylation analysis by direct bisulfite pyrosequencing in white blood cells. Mean methylation value for each CpG analysed by direct bisulfite pyrosequencing in DNA from white blood cells. Position of each CpG is indicated (TSS = +1). The mean methylation values for HSIL and SCC in LBC samples were added as a comparison. WBC, white blood cells.

We then used direct BP analysis to confirm the methylation status for the CpG located in *UTF1* promoter. We set a cut-off value of 20%, meaning that CpG methylation above this limit was considered as hypermethylated, whereas below 20% was considered as hypomethylated [27], [29]. We first validated our results by analyzing the same samples that were used for microarray analysis. We observed that UTF1 promoter was hypomethylated in normal Ecto, whereas the same sequence was demonstrated as totally hypermethylated in SCC. (Figure S3 and Table S2). To confirm *UTF1* promoter methylation signature, we performed direct BP on DNA isolated from liquid-based cytological (LBC) of four groups of samples: Ecto, LSIL, HSIL and SCC. We applied the same 20% cut-off as for DNA extracted from frozen tissues. In Ecto and LSIL, CpG were mostly hypomethylated and there was no difference in CpG methylation levels between these two groups (Figure 2A, 2B and Table S3, *p* = NS). There was a strong hypermethylation of *UTF1* promoter in HSIL and all the CpG in SCC samples were considered as totally hypermethylated (Figure 2A and 2B). Moreover, in SCC, all the CpG were significantly much more methylated than in Ecto and LSIL samples (Table S3, *p*<0.001, Ecto and LSIL *vs* SCC). Interestingly, CpG methylation levels in SCC were also significantly higher compared to HSIL (Table S3, for each CpG analyzed, *p* at least <0.05, SCC *vs* HSIL). Because LBC samples usually consist of heterogeneous mixtures of abnormal and normal squamous cells, as well as of inflammatory cells [33], we also analyzed the methylation status of *UTF1* promoter in DNA of 10 samples of white blood cells (WBC). As shown in Figure 3 and Table S4, in all WBC conditions, the promoter of *UTF1* appeared to be totally hypomethylated, with CpG methylation values ranging from 3 to 11 %, suggesting that *UTF1* methylation profile observed in LBC samples is not related to inflammatory cells.

### Expression profile of UTF1 during cervical carcinogenesis

**Figure 4:**
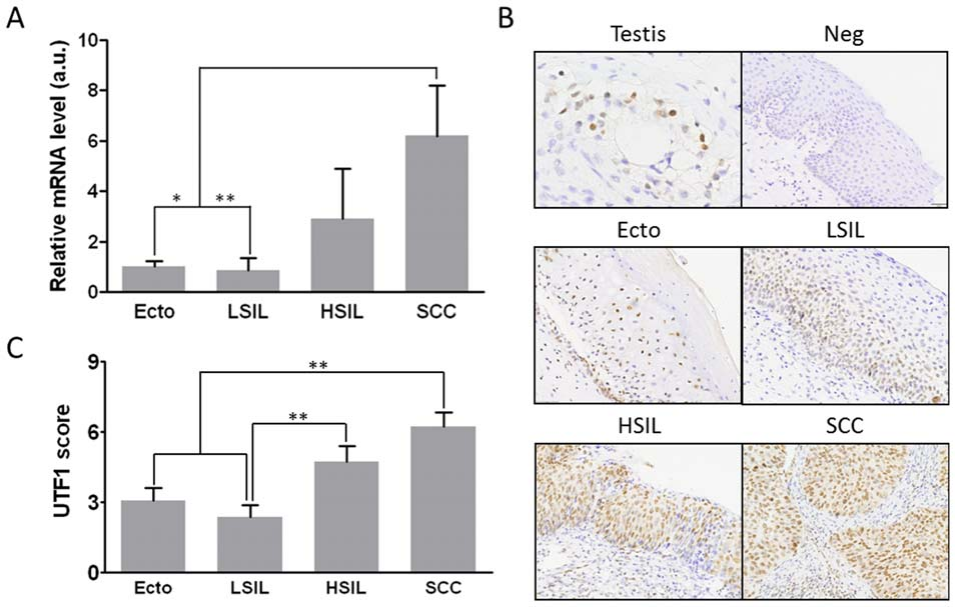
*UTF1* expression increases during cervical carcinogenesis. A) Expression of *UTF1* mRNA in liquid-based cytological samples. Expression was assessed by quantitative RT-PCR. Ecto, normal ectocervix; LSIL, low grade squamous intraepithelial lesion; HSIL, high grade squamous intraepithelial lesion; SCC, squamous cell carcinoma. B) Immunohistochemical staining of UTF1 protein in cervical tissue samples, magnification ×200. C) semi-quantitative analysis of staining. Testis, positive control; Neg, negative control without primary antibody. *, *p*<0.05; **, *p*<0.01 (Kruskal-Wallis followed by Dunn's Multiple Comparison test).

We then analyzed the expression of *UTF1* mRNA by quantitative RT-PCR in the same LBC samples. As shown in Figure 4A, in Ecto and LSIL, *UTF1* mRNA were similar (*p* = NS). Its expression increased in HSIL but not significantly. On the contrary, SCC expressed significantly more *UTF1* mRNA than in Ecto and LSIL (more than 6 fold, *p*<0.05 and *p*<0.01, respectively). A significant positive correlation was observed between *UTF1* mRNA expression and *UTF1* mean CpG methylation (Spearman correlation test, p = 0.0003, Figure S4).

*UTF1* expression was then analyzed by immunohistochemistry on a TMA dedicated to cervical carcinogenesis (18 Ecto, 11 LSIL, 18 HSIL and 19 SCC) with a previously described mouse monoclonal antibody [19], [21]. Among the 18 Ecto, Weak UTF1 staining was observed in the cell nuclei of the basal layer and of the epithelium in 90% of the samples, whereas it was not expressed by the remaining 10% samples (Figure 4B). The staining for UTF1 in LSIL was localized in the first third of the epithelium in all the samples tested and was similar to that observed in Ecto (Figure 4B and 4C, *p* = NS, LSIL *vs* Ecto). In contrast, immunoreactivity of UTF1 was much stronger and found in all cells of 92% of the HSIL samples, whereas it was not expressed by only one case (Figure 4B and 4C, *p*<0.05 *vs* LSIL). In SCC, staining of UTF1 was significantly stronger than in Ecto and LSIL in 75% of the samples (14 cases) (Figure 4B and 4C, *p*<0.01 *vs* Ecto and LSIL) and was expressed by all the cells within tumor samples.

### Expression of UTF1 and its regulators Oct4A and Sox2 in cancer cell lines

**Figure 5:**
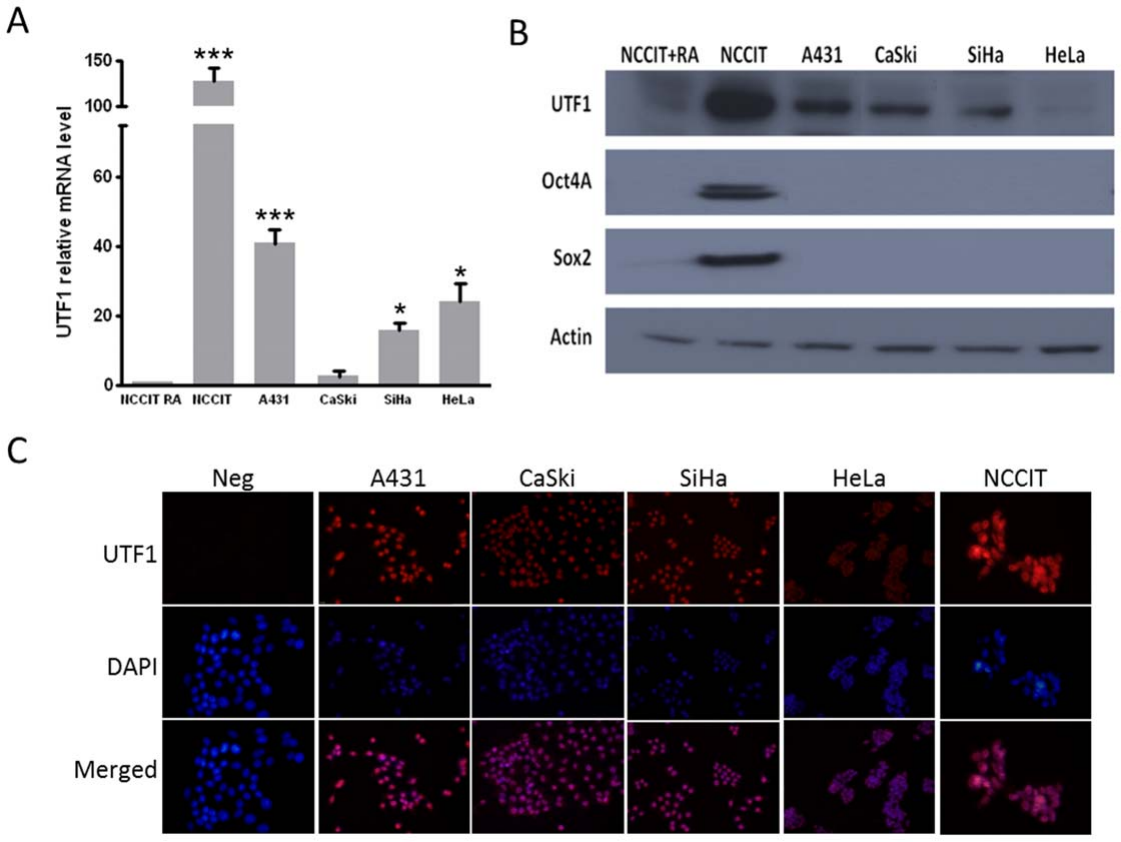
Expression of *UTF1* and its regulators *Oct4A* and *Sox2* in cervical cancer cell lines. A) Quantitative RT-PCR. B) Western Blot. C) Immunofluorescence; magnification ×400. Results are representative of 3 independent experiments. *, *p*<0.05 *vs* NCCIT+RA; ***, *p*<0.001 *vs* NCCIT+RA (Unpaired t test).

We next studied the expression of *UTF1* mRNA and protein in several cell lines derived from the anogenital area (A431, SiHa, CaSki and HeLa). The expression of *UTF1* mRNA was compared to that of the NCCIT carcinoma cell line which express high level of UTF1 while it was very low when these cells are treated with retinoic acid (RA) [18]. As shown in Figure 5A, in cancer cell lines, except for CasKi cells, *UTF1* mRNA was more expressed, than in NCCIT differentiated with RA (*p*<0.001 *vs* NCCIT+RA). The same profile of *UTF1* mRNA expression in cell lines was confirmed by end-point RT-PCR (Figure S1A), using previously published primers [21].

At the protein level, UTF1 was found in all cell lines, except that it was detected in CaSki and not in HeLa cells, suggesting post-transcriptional regulation of *UTF1* expression (Figure 5B). The specificity of the mouse monoclonal antibody was validated by overexpressing UTF1 with a plasmid containing *UTF1* human cDNA in SiHa and A431 cells (Figure S1B). We also analyzed the expression of the known regulators of *UTF1* expression and showed that neither Oct4 nor Sox2 were expressed in any cell lines (Figure 5B). The nuclear localization of UTF1 was confirmed by immunofluorescence in the cell lines (Figure 5C).

### Effect of 5-aza-deoxycytidine on cell proliferation and viability

**Figure 6:**
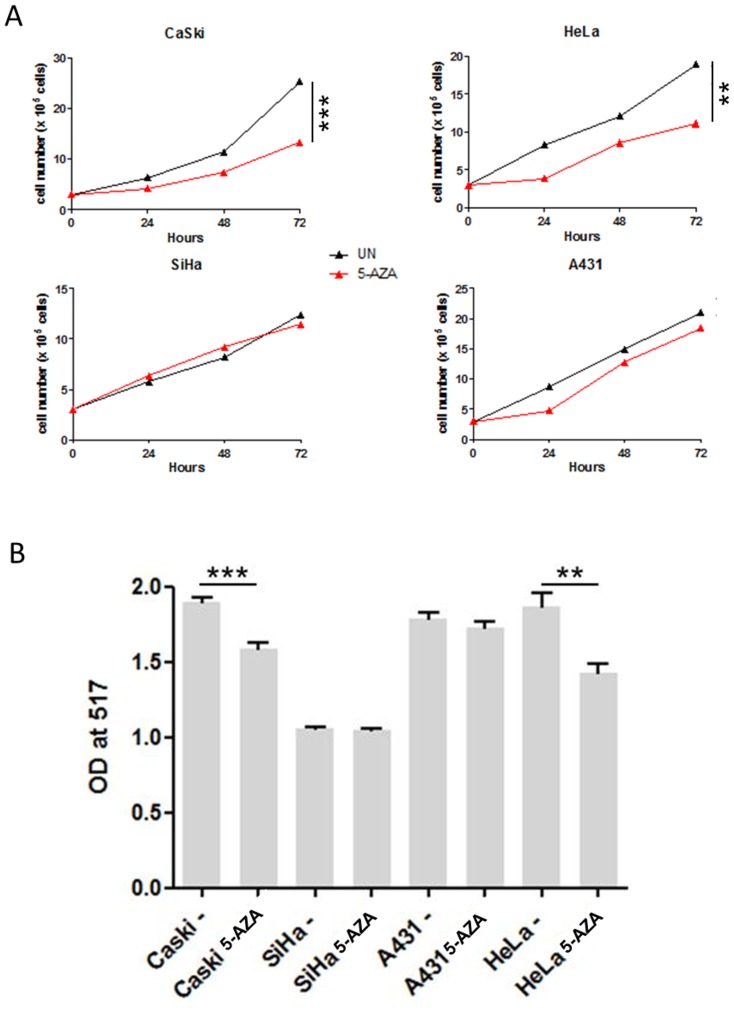
Effect of 5-aza on cell proliferation and viability. A) Cell proliferation. Cells were counted every 24 hrs during treatment with 5-AZA. B) Cell viability was measured with MTT test. **, p<0,01; ***, p<0,005 (Mann-Whitney U-Test). Results are representative of 3 independent experiments.

To assess a potential link between *UTF1* promoter methylation and its expression, cervical cell lines were treated with 5-aza. We first analyzed the impact of 5-aza on cell proliferation and viability. As shown on Figure 6A, 5-aza induced a decrease in HeLa and CaSki cells proliferation (p<0.01 and p<0.001 vs untreated cells, respectively), whereas it had no significant impact on SiHa and A431 cells (p = NS). Cell viability in response to 5-aza was assessed with MTT test. Similar results were obtained, with a decrease of cell viability observed only in HeLa and CaSki cells (Figure 6B).

### Methylation and expression of UTF1 after treatment with 5-aza-2′-deoxycytidine

**Figure 7:**
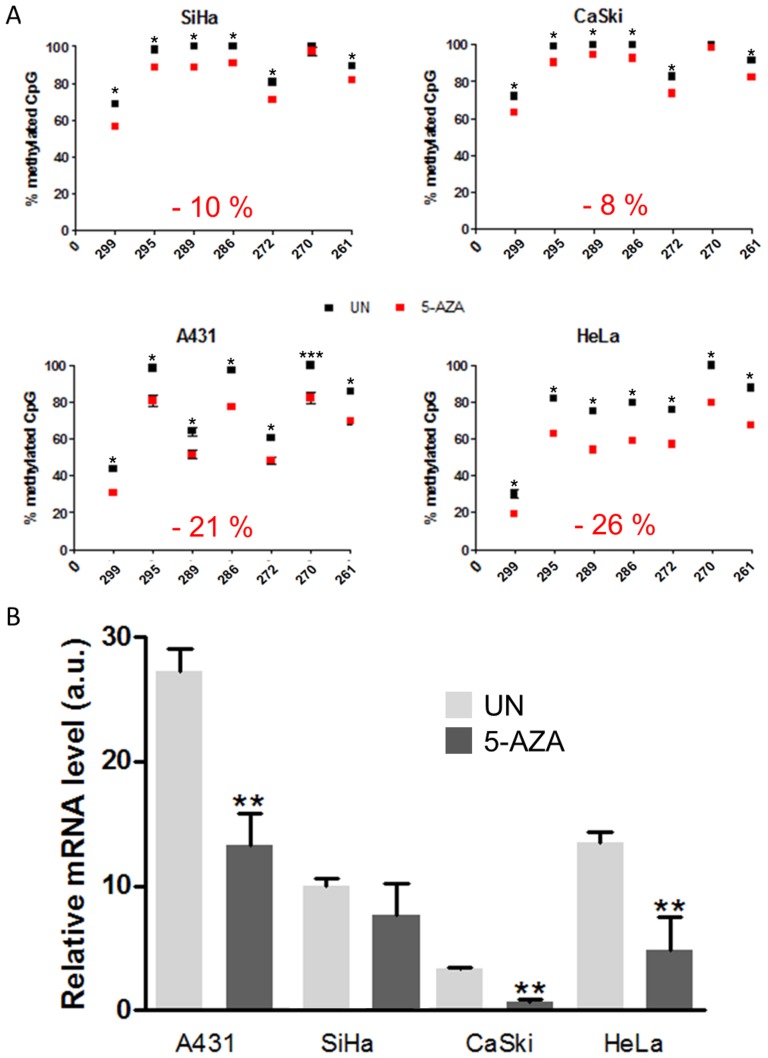
Effect of 5-aza on *UTF1* promoter methylation and expression in epithelial cancer cell lines. A) BP analysis of *UTF1* methylation status in cancer cell lines derived from the anogenital area with (5-AZA) or without (UN) 5-AZA for 72 hrs. B) Quantitative RT-PCR of cancer cell lines derived from the anogenital sphere with (5-AZA) or without (UN) 5-AZA for 72 hrs. Results are representative of 3 independent experiments. **, *p*<0.01 *vs* UN (Mann-Whitney U Test).

After treatment with 5-aza, UTF1 promoter methylation was checked by BP. As shown in Figure 7A, UTF1 promoter appeared to be heavily methylated in all the untreated cell lines tested, with global methylation value being 92% for CaSki, 91 % for SiHa, 79 % for A431 and 76 % for HeLa. Treatment with 5-aza decreased the methylation of the CpG with varying efficacy. In SiHa and CaSki global methylation was decreased of 10% and 8% respectively (Figure 7A, see values in red on graph). On the contrary, the loss of methylation was stronger in A431 and HeLa cell lines (−21% and −26%, respectively). At the mRNA level (Figure 7B), treatment with 5-aza inhibited UTF1 expression in 3 of the 4 cell lines, excepted in SiHa were UTF1 mRNA expression remained unchanged. In order to unravel a possible link between UTF1 promoter methylation and expression, we tried to correlate the global methylation and the mRNA levels in the four cell lines. A significant positive correlation was observed between UTF1 mRNA expression and UTF1 mean CpG methylation in A431 and HeLa cell lines (Spearman correlation test, p = 0.0167 and p = 0.0333, respectively, Figure S5), but not in SiHa and CaSki (p = 0.8438 and p = 0.0598, respectively, Figure S5).

## Discussion

Cervical cancer development can take decades. Therefore adequate diagnosis and treatment of preneoplastic lesions might decrease its rate of development [2], [3]. It is well established that DNA methylation patterns could be used to improve cancer diagnosis and/or prognosis [34]. However, in spite of clinical research progress, there is still no reliable epigenetic biomarker for cervical cancer diagnosis [35]. Currently, methylation specific PCR (MSP) is the gold standard to identify CpG hypermethylation. However it can lead to false positive results and the methylation status of only one CpG can be assessed in each PCR reaction [34]. Reports have already evaluated promoter methylation by MSP in Papanicolaou smears, which contain a mixed cell population, thus confirming the usefulness of these samples for the identification of putative epigenetic biomarkers [36], [37]. Other commercialized techniques, such as Methylight or quantitative MSP, were also used to identify promoter methylation profile in DNA from liquid-based cytological (LBC) samples. These techniques allowed to discriminate between normal cytological samples and HSIL [38], [39]. In this study we performed direct bisulfite pyrosequencing (BP) which was reported to give accurate and reproducible results for up to 20 CpG within up 120 nucleotides [34], [40]. The data obtained in this study were far below these limits since the pyrosequenced fragment was of small size (50 bp) and contained only 8 CpG, thus reducing the risk for loss of sensitivity or specificity [34].

Direct BP of LBC samples has already been used to assess methylation status of HPV DNA in cervical lesions. The authors were able to demonstrate that CpG methylation status within the upstream regulatory region of *L1* gene allowed to discriminate between CIN3 and CIN2 or lower grade lesions [41]. To our knowledge, the current study is the first one to show that methylation profile of non viral promoter gene can be assessed by direct BP on LBC samples. With this technique, we showed that *UTF1* hypermethylation status might be a useful biomarker to discriminate normal Ecto and LSIL from HSIL and SCC and to improve cervical cancer diagnosis.

The specificity of stem cell (SC) markers such as UTF1, Oct4A, Sox2 or KLF4 is well established for embryonic or induced pluripotent SC [42]. In the tumor context, cancer SC (CSC) have been proposed to be involved in resistance to therapy and metastasis formation [5], [43]. However the involvement of SC markers and their epigenetic regulation during epithelial carcinogenesis are still poorly defined. This is in part due to the rarity of CSC and to the phenotypic cell heterogeneity within tumor mass [5], [44].

Here, we demonstrated the presence of UTF1 in normal and tumor squamous epithelial tissues, as well as in cancer cell lines. The *UTF1* expression pattern that we observed did not fit the definition of CSC which account for a very small proportion of cells within tumor mass [6], [44]. This hypothesis is supported by the work of Ji et al. showing that *Sox2* expression increases during cervical carcinogenesis which is consistent with the expression profile of *UTF1* [16]. This also in agreement with a very recent work proposing that *UTF1* expression in adult tissue may have different roles than those observed stem cell regulation at the earliest stages of embryogenesis [24]. In addition, we did not detect any expression of *Oct4A* in cell lines, which is in agreement with other studies conducted in breast and in cerebral tumors [31], [45]. It has been also previously described that UTF1 depletion decreases the tumorigenicity of teratoma subcutaneously injected in nude mice [46]. In some LSIL and SCC samples, a cytoplasmic staining of UTF1 was also observed, which is in agreement with a recent work investigating skin keratinocytes [24]. One possible explanation could be related to a role of UTF1 in cell division as it was proposed for the *B lymphoma Mo-MLV insertion region 1 homolog* (*BMI-1*) gene in skin cancer but the mechanism behind these observations is still unknown [47]. Therefore, we can postulate that *UTF1* upregulation by itself might participate to the global gene expression alterations leading to cancer development by locking tumor-specific gene expression pattern through its fixation to chromatin [18], [48]. This mechanism could be mediated by estrogens, which were shown to increase DNA methyltransferase activity or cervical carcinogenesis [49], [50]. This is supported by the fact that, in endometriosis, an estrogen-dependent disease characterized by the presence of endometrium outside the uterine cavity, higher levels of *UTF1* mRNA were found in comparison with normal endometrium [51]. Consequently, in response to microenvironment or intracellular signaling, *UTF1* might be overexpressed, participate in epigenetic cellular reprogramming and confer preneoplastic cells a proliferating advantage. This would ultimately lead to cancer cell engraftment and growth, in agreement with its known role as an enhancer of colony formation of induced pluripotent SC [42].

The treatment with 5-aza induced a global decrease of 21% and 26% of *UTF1* promoter in A431 and HeLa cells, respectively. This range of variation in response to 5-aza has been previously described for other genes such as *somatostatin* in gastric cancer cell lines, *GADD45A* in osteosarcoma cell lines or *LCR* and *L1* of HPV-16 in cervical cancer cell line [52]–[54].The correlation between *UTF1* expression and promoter methylation was also unexpected. However, similar results have already been reported. For example, in healthy tissue, the profiling of DNA methylation on chromosomes 6, 20 and 22 showed that, for 63% of the genes tested, DNA methylation was not related to inhibition of expression [55]. In endometrial cancer, *survivin* promoter hypermethylation of CpG within transcriptional repressor binding sites was correlated with its upregulation [56]. In the same way, in breast cancer samples, the promoter of the *KLHDC7B* gene was hypermethylated and associated with its overexpression in comparison with normal mammary tissue [57]. *In vitro*, the methylation of the human glycoprotein hormone α-subunit (*GPHα*) gene was found to be hypermethylated in cell lines producing high amount of this protein, whereas low level were detected in cell lines with hypomethylated *GPHα* gene sequence [58].

Our results suggest that, in the absence of Oct4A and Sox2, *UTF1* promoter hypermethylation might also participate in gene expression. This is supported by the methylation status of *UTF1* promoter in NCCIT cells differentiated or not with retinoic acid which appear totally hypomethylated in both conditions (Figure S6). In NCCIT UN, *UTF1* expression is stimulated by Oct4a and Sox2 (Fig. 5B and [21], [22]), whereas in NCCIT differentiated with RA, UTF1, Oct4a and Sox2 are not expressed (Figure 5B and [18], [22]). On the contrary, in untreated epithelial cancer cell lines, *UTF1* promoter appears to be heavily methylated and expressed in spite of the absence of Oct4a and Sox2. This strongly suggests that *UTF1* promoter methylation is involved in the control of its expression in the absence of its two well described regulators. This is also corroborated by the positive correlation between *UTF1* methylation and expression in 2 cell lines (A431 and HeLa) of the 4 that were treated with 5-aza, with a third cell line (CaSki) being near significance. However the molecular mechanism involved need to be elucidated and might be cell-specific as showed by the lack of effect of 5-aza in SiHa cells. The p53 or Sp1 transcription factors might participate in this pathway. Indeed p53 protein is able to inhibit *survivin* expression when its promoter is demethylated [56]. This is supported by the fact that HeLa cells express higher level of p53 than SiHa cells [59]. In osteoblastic cells the binding of Sp1 and its transcriptional activity were demonstrated to be enhanced when *podoplanin* promoter was hypermethylated [60].

In conclusion, we demonstrated that *UTF1* promoter hypermethylation is associated with its overexpression during cervical carcinogenesis, suggesting that *UTF1* promoter methylation profile might be a useful diagnosis biomarker. We also showed that, *in vitro*, *UTF1* expression is not restricted to SC and that it is localized in the nucleus of somatic tumor cells independently of Oct4A and Sox2 expression.

## Supporting Information

Figure S1 **Validation of UTF1 mRNA expression and UTF1 antibody specificity.** A) RT-PCR of UTF1 performed with previously described UTF1 primers (see ref [21]). B) Western Blot of A431 and SiHa cell lines transiently transfected with either empty plasmid (PCDNA.3) or plasmid containing UTF1 cDNA (UTF1). NCCIT UN and NCCIT RA are positive and negative controls for UTF1 expression, respectively.
(TIF)

Figure S2 **Structure of** ***UTF1*** **gene.** Red triangles, CpG analysed by microarray; black box, DNA sequence analysed by direct bisulfite pyrosequencing; blue and green circles, binding sites for Sox2 and Oct4A.
(TIF)

Figure S3 **Validation of** ***UTF1*** **promoter methylation by direct bisulfite pyrosequencing in samples used for microarray screening.** A) mean value of methylation for each CpG analysed. B) CpG methylation in each samples. A cut-off value was set at 20%, meaning that value above 20% is considered as hypermethylated, whereas above means hypomethylated. Position of each CpG is indicated (TSS = +1). In red is indicated the Cpg analysed on Illumina chip. White circle, hypomethylated CpG; black circle, hypermethylated CpG.
(TIF)

Figure S4 **Correlation between** ***UTF1*** **promoter mean CpG methylation and UTF1 mRNA expression.** The mean of CpG methylation was calculated for each sample. Data from qPCR were log transformed before being plotted. Correlation between *UTF1* mean CpG methylation and mRNA expression was evaluated by Spearman's rank correlation test, reported p-value was two-sided.
(TIF)

Figure S5 **Correlation between** ***UTF1*** **promoter mean CpG methylation and UTF1 mRNA expression in cancer cell lines treated or not with 5-AZA.** The mean of CpG methylation was calculated for each sample. Correlation between UTF1 mean CpG methylation and mRNA expression was evaluated by Spearman's rank correlation test, reported p-value was two-sided.
(TIF)

Figure S6 ***UTF1*** **promoter methylation analysis by direct bisulfite pyrosequencing in NCCIT and NCCIT differentiated with retinoic acid (NCCIT RA).** Position of each CpG is indicated (TSS = +1). For comparison, methylation values for epithelial cancer cell lines used in this work were added.
(TIF)

Table S1 The β-values for each CpG of stem cell genes analysed by microarray.
(PDF)

Table S2 Values of *UTF1* CpG methylation in DNA from frozen tissue by direct bisulfite pyrosequencing.
(PDF)

Table S3 Values of *UTF1* CpG methylation in DNA from liquid-based cytological samples by direct bisulfite pyrosequencing.
(PDF)

Table S4 Values of *UTF1* CpG methylation in DNA from white blood cells by direct bisulfite pyrosequencing.
(PDF)

## References

[1] Ferlay J, Shin HR, Bray F, Forman D, Mathers C, et al (2010) Estimates of worldwide burden of cancer in 2008: GLOBOCAN 2008. International journal of cancer Journal international du cancer 127: 2893–2917. 21351269 10.1002/ijc.25516

[2] zur Hausen H (2009) Papillomaviruses in the causation of human cancers - a brief historical account. Virology 384: 260–265. 19135222 10.1016/j.virol.2008.11.046

[3] Hall S, Lorincz A, Shah F, Sherman ME, Abbas F, et al (1996) Human papillomavirus DNA detection in cervical specimens by hybrid capture: correlation with cytologic and histologic diagnoses of squamous intraepithelial lesions of the cervix. Gynecologic oncology 62: 353–359. 8812532 10.1006/gyno.1996.0248

[4] Chik F, Szyf M, Rabbani SA (2011) Role of epigenetics in cancer initiation and progression. Advances in experimental medicine and biology 720: 91–104. 21901621 10.1007/978-1-4614-0254-1_8

[5] Hanahan D, Weinberg RA (2011) Hallmarks of cancer: the next generation. Cell 144: 646–674. 21376230 10.1016/j.cell.2011.02.013

[6] Reya T, Morrison SJ, Clarke MF, Weissman IL (2001) Stem cells, cancer, and cancer stem cells. Nature 414: 105–111. 11689955 10.1038/35102167

[7] Mathieu J, Zhang Z, Zhou W, Wang AJ, Heddleston JM, et al (2011) HIF induces human embryonic stem cell markers in cancer cells. Cancer research 71: 4640–4652. 21712410 10.1158/0008-5472.CAN-10-3320 PMC3129496

[8] Feng D, Peng C, Li C, Zhou Y, Li M, et al (2009) Identification and characterization of cancer stem-like cells from primary carcinoma of the cervix uteri. Oncology reports 22: 1129–1134. 19787230 10.3892/or_00000545

[9] Bortolomai I, Canevari S, Facetti I, De Cecco L, Castellano G, et al (2010) Tumor initiating cells: development and critical characterization of a model derived from the A431 carcinoma cell line forming spheres in suspension. Cell cycle 9: 1194–1206. 20237414 10.4161/cc.9.6.11108

[10] Liu D, Zhou P, Zhang L, Wu G, Zheng Y, et al (2011) Differential expression of Oct4 in HPV-positive and HPV-negative cervical cancer cells is not regulated by DNA methyltransferase 3A. Tumour biology : the journal of the International Society for Oncodevelopmental Biology and Medicine 32: 941–950. 21674242 10.1007/s13277-011-0196-z

[11] Mueller T, Luetzkendorf J, Nerger K, Schmoll HJ, Mueller LP (2009) Analysis of OCT4 expression in an extended panel of human tumor cell lines from multiple entities and in human mesenchymal stem cells. Cellular and molecular life sciences : CMLS 66: 495–503. 19023518 10.1007/s00018-008-8623-z PMC11131475

[12] Wang X, Dai J (2010) Concise review: isoforms of OCT4 contribute to the confusing diversity in stem cell biology. Stem cells 28: 885–893. 20333750 10.1002/stem.419 PMC2962909

[13] Pain D, Chirn GW, Strassel C, Kemp DM (2005) Multiple retropseudogenes from pluripotent cell-specific gene expression indicates a potential signature for novel gene identification. The Journal of biological chemistry 280: 6265–6268. 15640145 10.1074/jbc.C400587200

[14] Pellacani D, Packer RJ, Frame FM, Oldridge EE, Berry PA, et al (2011) Regulation of the stem cell marker CD133 is independent of promoter hypermethylation in human epithelial differentiation and cancer. Molecular cancer 10: 94. 21801380 10.1186/1476-4598-10-94 PMC3162587

[15] Shmelkov SV, Butler JM, Hooper AT, Hormigo A, Kushner J, et al (2008) CD133 expression is not restricted to stem cells, and both CD133+ and CD133− metastatic colon cancer cells initiate tumors. The Journal of clinical investigation 118: 2111–2120. 18497886 10.1172/JCI34401 PMC2391278

[16] Ji J, Zheng PS (2010) Expression of Sox2 in human cervical carcinogenesis. Human pathology 41: 1438–1447. 20709360 10.1016/j.humpath.2009.11.021

[17] Xuan YH, Jung HS, Choi YL, Shin YK, Kim HJ, et al (2006) Enhanced expression of hedgehog signaling molecules in squamous cell carcinoma of uterine cervix and its precursor lesions. Modern pathology : an official journal of the United States and Canadian Academy of Pathology, Inc 19: 1139–1147. 10.1038/modpathol.3800600 16778829

[18] Kooistra SM, Thummer RP, Eggen BJ (2009) Characterization of human UTF1, a chromatin-associated protein with repressor activity expressed in pluripotent cells. Stem cell research 2: 211–218. 19393592 10.1016/j.scr.2009.02.001

[19] Wang P, Li J, Allan RW, Guo CC, Peng Y, et al (2010) Expression of UTF1 in primary and metastatic testicular germ cell tumors. American journal of clinical pathology 134: 604–612. 20855642 10.1309/AJCPB44HBKINJNYU

[20] van den Boom V, Kooistra SM, Boesjes M, Geverts B, Houtsmuller AB, et al (2007) UTF1 is a chromatin-associated protein involved in ES cell differentiation. The Journal of cell biology 178: 913–924. 17785516 10.1083/jcb.200702058 PMC2064617

[21] Kristensen DM, Nielsen JE, Skakkebaek NE, Graem N, Jacobsen GK, et al (2008) Presumed pluripotency markers UTF-1 and REX-1 are expressed in human adult testes and germ cell neoplasms. Human reproduction 23: 775–782. 18281244 10.1093/humrep/den010

[22] Imamura M, Miura K, Iwabuchi K, Ichisaka T, Nakagawa M, et al (2006) Transcriptional repression and DNA hypermethylation of a small set of ES cell marker genes in male germline stem cells. BMC developmental biology 6: 34. 16859545 10.1186/1471-213X-6-34 PMC1564388

[23] Melone MA, Giuliano M, Squillaro T, Alessio N, Casale F, et al (2009) Genes involved in regulation of stem cell properties: studies on their expression in a small cohort of neuroblastoma patients. Cancer biology & therapy 8: 1300–1306. 19458492 10.4161/cbt.8.13.8890

[24] Reinisch CM, Mildner M, Petzelbauer P, Pammer J (2011) Embryonic stem cell factors undifferentiated transcription factor-1 (UFT-1) and reduced expression protein-1 (REX-1) are widely expressed in human skin and may be involved in cutaneous differentiation but not in stem cell fate determination. International journal of experimental pathology 92: 326–332. 21446939 10.1111/j.1365-2613.2011.00769.x PMC3193146

[25] Arafa M, Boniver J, Delvenne P (2008) Detection of HPV-induced cervical (pre) neoplastic lesions: a tissue microarray (TMA) study. Applied immunohistochemistry & molecular morphology : AIMM/official publication of the Society for Applied Immunohistochemistry 16: 422–432. 10.1097/PAI.0b013e318166fd42 18542030

[26] Gunderson KL, Bibikova M, Le J, Barnes B, Saedinia-Melnyk S, et al (2009) Genome-wide DNA methylation profiling using Infinium (R) assay. Epigenomics 1: 177–200. 22122642 10.2217/epi.09.14

[27] Gronniger E, Weber B, Heil O, Peters N, Stab F, et al (2010) Aging and chronic sun exposure cause distinct epigenetic changes in human skin. PLoS genetics 6: e1000971. 20523906 10.1371/journal.pgen.1000971 PMC2877750

[28] de Hoon MJ, Imoto S, Nolan J, Miyano S (2004) Open source clustering software. Bioinformatics 20: 1453–1454. 14871861 10.1093/bioinformatics/bth078

[29] Takahashi Y, Iwai M, Kawai T, Arakawa A, Ito T, et al (2011) Aberrant expression of tumor suppressors CADM1 and 4.1B in invasive lesions of primary breast cancer. Breast cancer doi: 10.1007/s12282-011-0272-7. 10.1007/s12282-011-0272-7 21526423

[30] Herfs M, Hubert P, Suarez-Carmona M, Reschner A, Saussez S, et al (2010) Regulation of p63 isoforms by snail and slug transcription factors in human squamous cell carcinoma. The American journal of pathology 176: 1941–1949. 20150431 10.2353/ajpath.2010.090804 PMC2843482

[31] Zhao S, Yuan Q, Hao H, Guo Y, Liu S, et al (2011) Expression of OCT4 pseudogenes in human tumours: lessons from glioma and breast carcinoma. The Journal of pathology 223: 672–682. 21341266 10.1002/path.2827

[32] Mosmann T (1983) Rapid colorimetric assay for cellular growth and survival: application to proliferation and cytotoxicity assays. J Immunol Methods 65: 55–63. 6606682 10.1016/0022-1759(83)90303-4

[33] Kahn SL, Ronnett BM, Gravitt PE, Gustafson KS (2008) Quantitative methylation-specific PCR for the detection of aberrant DNA methylation in liquid-based Pap tests. Cancer 114: 57–64. 18181097 10.1002/cncr.23258 PMC2648805

[34] Dehan P, Kustermans G, Guenin S, Horion J, Boniver J, et al (2009) DNA methylation and cancer diagnosis: new methods and applications. Expert review of molecular diagnostics 9: 651–657. 19817550 10.1586/erm.09.53

[35] Wentzensen N, Sherman ME, Schiffman M, Wang SS (2009) Utility of methylation markers in cervical cancer early detection: appraisal of the state-of-the-science. Gynecologic oncology 112: 293–299. 19054549 10.1016/j.ygyno.2008.10.012 PMC2673716

[36] Kim JH, Choi YD, Lee JS, Lee JH, Nam JH, et al (2010) Assessment of DNA methylation for the detection of cervical neoplasia in liquid-based cytology specimens. Gynecologic oncology 116: 99–104. 19836067 10.1016/j.ygyno.2009.09.032

[37] Feng Q, Balasubramanian A, Hawes SE, Toure P, Sow PS, et al (2005) Detection of hypermethylated genes in women with and without cervical neoplasia. Journal of the National Cancer Institute 97: 273–282. 15713962 10.1093/jnci/dji041

[38] Overmeer RM, Henken FE, Bierkens M, Wilting SM, Timmerman I, et al (2009) Repression of MAL tumour suppressor activity by promoter methylation during cervical carcinogenesis. The Journal of pathology 219: 327–336. 19662663 10.1002/path.2598

[39] Lim EH, Ng SL, Li JL, Chang AR, Ng J, et al (2010) Cervical dysplasia: assessing methylation status (Methylight) of CCNA1, DAPK1, HS3ST2, PAX1 and TFPI2 to improve diagnostic accuracy. Gynecologic oncology 119: 225–231. 20708786 10.1016/j.ygyno.2010.07.028

[40] Dupont JM, Tost J, Jammes H, Gut IG (2004) De novo quantitative bisulfite sequencing using the pyrosequencing technology. Analytical biochemistry 333: 119–127. 15351288 10.1016/j.ab.2004.05.007

[41] Sun C, Reimers LL, Burk RD (2011) Methylation of HPV16 genome CpG sites is associated with cervix precancer and cancer. Gynecologic oncology 121: 59–63. 21306759 10.1016/j.ygyno.2011.01.013 PMC3062667

[42] Zhao Y, Yin X, Qin H, Zhu F, Liu H, et al (2008) Two supporting factors greatly improve the efficiency of human iPSC generation. Cell stem cell 3: 475–479. 18983962 10.1016/j.stem.2008.10.002

[43] Alison MR, Lim SM, Nicholson LJ (2011) Cancer stem cells: problems for therapy? The Journal of pathology 223: 147–161. 21125672 10.1002/path.2793

[44] Suva ML, Riggi N, Stehle JC, Baumer K, Tercier S, et al (2009) Identification of cancer stem cells in Ewing's sarcoma. Cancer research 69: 1776–1781. 19208848 10.1158/0008-5472.CAN-08-2242

[45] Cantz T, Key G, Bleidissel M, Gentile L, Han DW, et al (2008) Absence of OCT4 expression in somatic tumor cell lines. Stem cells 26: 692–697. 18032701 10.1634/stemcells.2007-0657

[46] Nishimoto M, Miyagi S, Yamagishi T, Sakaguchi T, Niwa H, et al (2005) Oct-3/4 maintains the proliferative embryonic stem cell state via specific binding to a variant octamer sequence in the regulatory region of the UTF1 locus. Molecular and cellular biology 25: 5084–5094. 15923625 10.1128/MCB.25.12.5084-5094.2005 PMC1140574

[47] Reinisch CM, Uthman A, Erovic BM, Pammer J (2007) Expression of BMI-1 in normal skin and inflammatory and neoplastic skin lesions. J Cutan Pathol 34: 174–180. 17244030 10.1111/j.1600-0560.2006.00587.x

[48] Kooistra SM, van den Boom V, Thummer RP, Johannes F, Wardenaar R, et al (2010) Undifferentiated embryonic cell transcription factor 1 regulates ESC chromatin organization and gene expression. Stem cells 28: 1703–1714. 20715181 10.1002/stem.497

[49] Zhao Z, Fan L, Frick KM (2010) Epigenetic alterations regulate estradiol-induced enhancement of memory consolidation. Proceedings of the National Academy of Sciences of the United States of America 107: 5605–5610. 20212170 10.1073/pnas.0910578107 PMC2851775

[50] Chung SH, Franceschi S, Lambert PF (2010) Estrogen and ERalpha: culprits in cervical cancer? Trends in endocrinology and metabolism: TEM 21: 504–511. 20456973 10.1016/j.tem.2010.03.005 PMC2914219

[51] Forte A, Schettino MT, Finicelli M, Cipollaro M, Colacurci N, et al (2009) Expression pattern of stemness-related genes in human endometrial and endometriotic tissues. Molecular medicine 15: 392–401. 19690622 10.2119/molmed.2009.00068 PMC2727462

[52] Kalantari M, Lee D, Calleja-Macias IE, Lambert PF, Bernard HU (2008) Effects of cellular differentiation, chromosomal integration and 5-aza-2′-deoxycytidine treatment on human papillomavirus-16 DNA methylation in cultured cell lines. Virology 374: 292–303. 18242658 10.1016/j.virol.2007.12.016 PMC2556224

[53] Jackson K, Soutto M, Peng D, Hu T, Marshal D, et al (2011) Epigenetic silencing of somatostatin in gastric cancer. Dig Dis Sci 56: 125–130. 20927589 10.1007/s10620-010-1422-z PMC3082506

[54] Al-Romaih K, Sadikovic B, Yoshimoto M, Wang Y, Zielenska M, et al (2008) Decitabine-induced demethylation of 5′ CpG island in GADD45A leads to apoptosis in osteosarcoma cells. Neoplasia 10: 471–480. 18472964 10.1593/neo.08174 PMC2373908

[55] Eckhardt F, Lewin J, Cortese R, Rakyan VK, Attwood J, et al (2006) DNA methylation profiling of human chromosomes 6, 20 and 22. Nature genetics 38: 1378–1385. 17072317 10.1038/ng1909 PMC3082778

[56] Nabilsi NH, Broaddus RR, Loose DS (2009) DNA methylation inhibits p53-mediated survivin repression. Oncogene 28: 2046–2050. 19363521 10.1038/onc.2009.62

[57] Kim TW, Kim YJ, Lee HJ, Min SY, Kang HS, et al (2010) Hs.137007 is a novel epigenetic marker hypermethylated and up-regulated in breast cancer. International journal of oncology 36: 1105–1111. 20372783 10.3892/ijo_00000592

[58] Cox GS, Gutkin DW, Haas MJ, Cosgrove DE (1998) Isolation of an Alu repetitive DNA binding protein and effect of CpG methylation on binding to its recognition sequence. Biochimica et biophysica acta 1396: 67–87. 9524225 10.1016/s0167-4781(97)00175-9

[59] Hietanen S, Lain S, Krausz E, Blattner C, Lane DP (2000) Activation of p53 in cervical carcinoma cells by small molecules. Proceedings of the National Academy of Sciences of the United States of America 97: 8501–8506. 10900010 10.1073/pnas.97.15.8501 PMC26977

[60] Hantusch B, Kalt R, Krieger S, Puri C, Kerjaschki D (2007) Sp1/Sp3 and DNA-methylation contribute to basal transcriptional activation of human podoplanin in MG63 versus Saos-2 osteoblastic cells. BMC molecular biology 8: 20. 17343736 10.1186/1471-2199-8-20 PMC1828165

