# Supplementary figures and images for: Aberrant Promoter Methylation and Expression of UTF1 during Cervical Carcinogenesis
Source: PLoS One. 2012 Aug 3;7(8):e42704. doi: 10.1371/journal.pone.0042704 (PMC3411846; doi:10.1371/journal.pone.0042704)

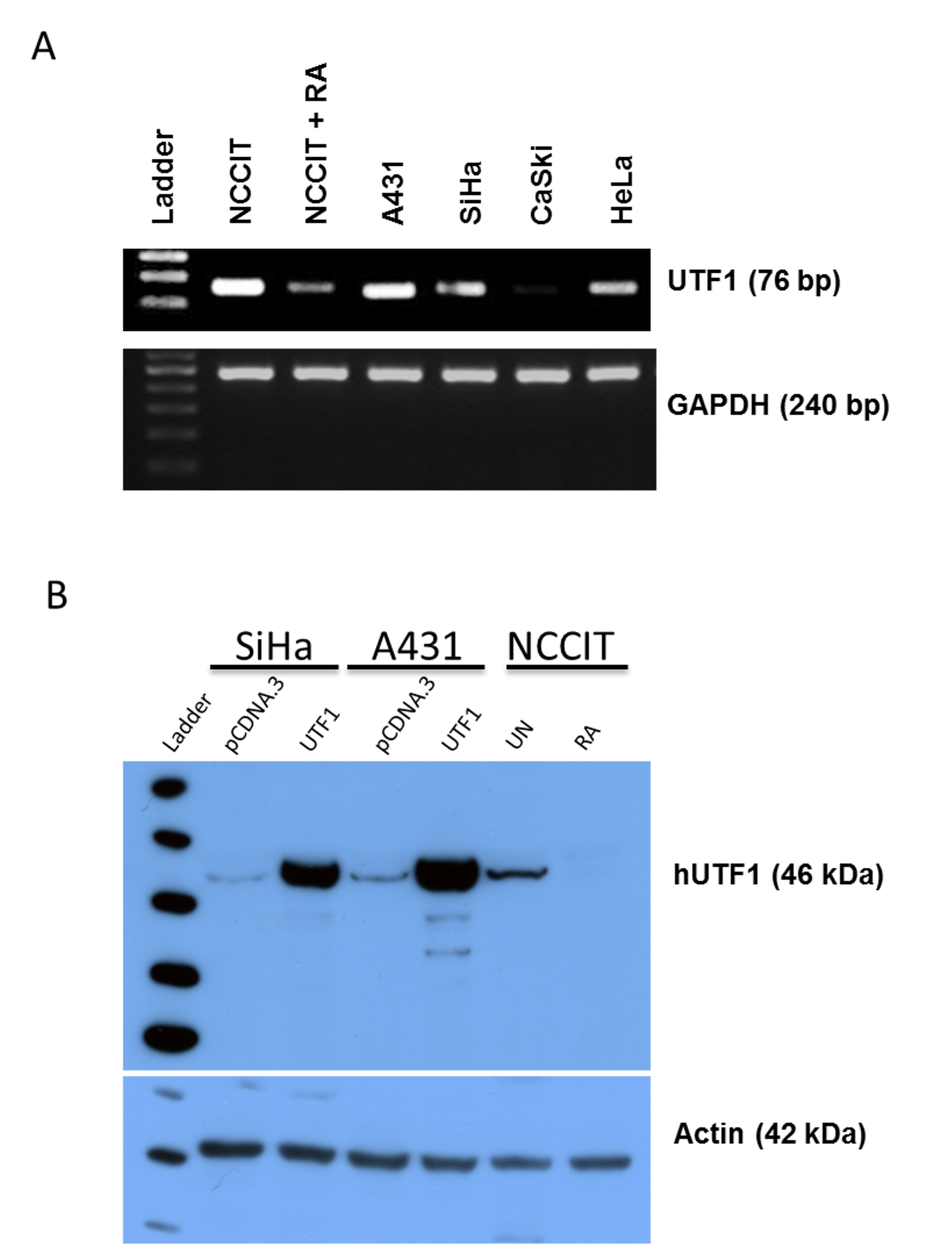

Supplement: Figure S1 — Validation of UTF1 mRNA expression and UTF1 antibody specificity. A) RT-PCR of UTF1 performed with previously described UTF1 primers (see ref [21]). B) Western Blot of A431 and SiHa cell lines transiently transfected with either empty plasmid (PCDNA.3) or plasmid containing UTF1 cDNA (UTF1). NCCIT UN and NCCIT RA are positive and negative controls for UTF1 expression, respectively. (TIF) [file pone.0042704.s001.tif]

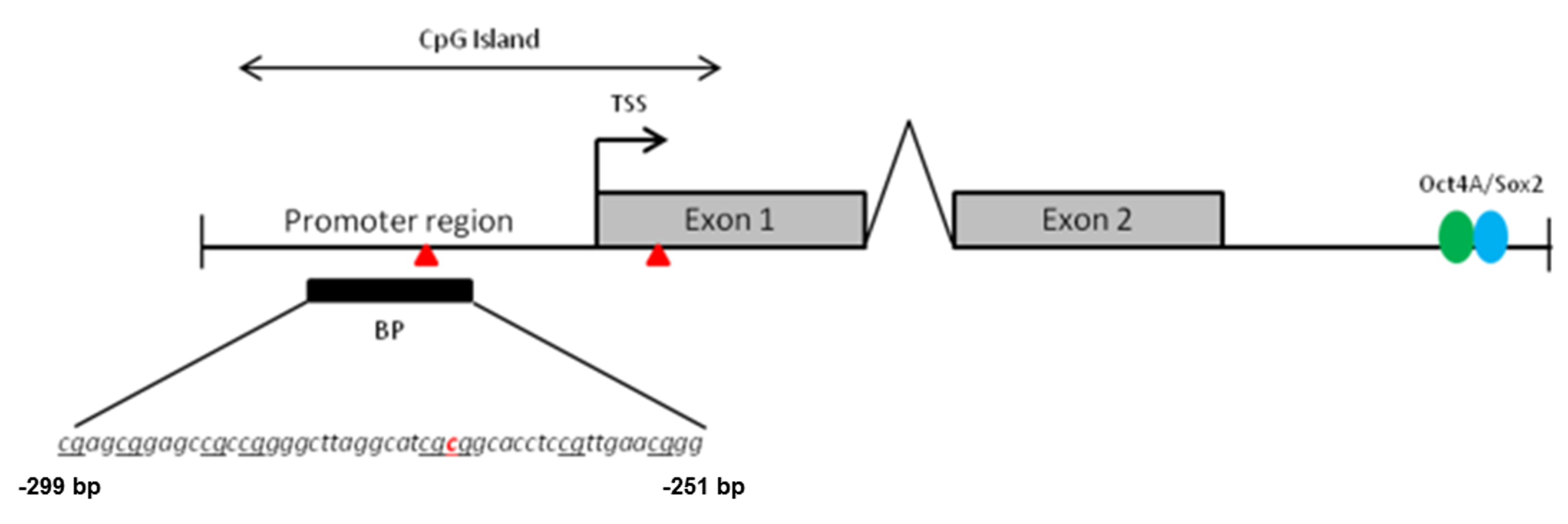

Supplement: Figure S2 — Structure of UTF1 gene. Red triangles, CpG analysed by microarray; black box, DNA sequence analysed by direct bisulfite pyrosequencing; blue and green circles, binding sites for Sox2 and Oct4A. (TIF) [file pone.0042704.s002.tif]

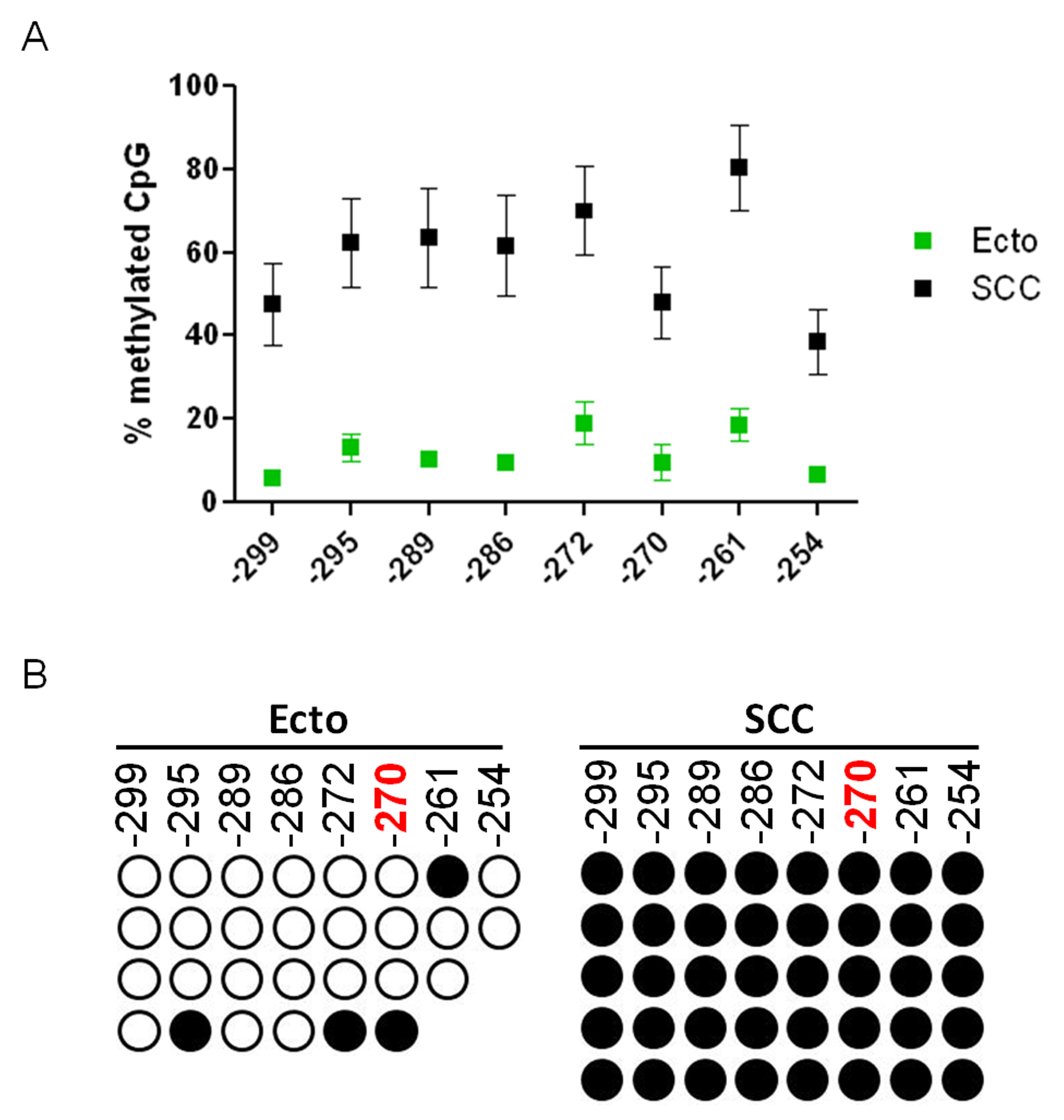

Supplement: Figure S3 — Validation of UTF1 promoter methylation by direct bisulfite pyrosequencing in samples used for microarray screening. A) mean value of methylation for each CpG analysed. B) CpG methylation in each samples. A cut-off value was set at 20%, meaning that value above 20% is considered as hypermethylated, whereas above means hypomethylated. Position of each CpG is indicated (TSS = +1). In red is indicated the Cpg analysed on Illumina chip. White circle, hypomethylated CpG; black circle, hypermethylated CpG. (TIF) [file pone.0042704.s003.tif]

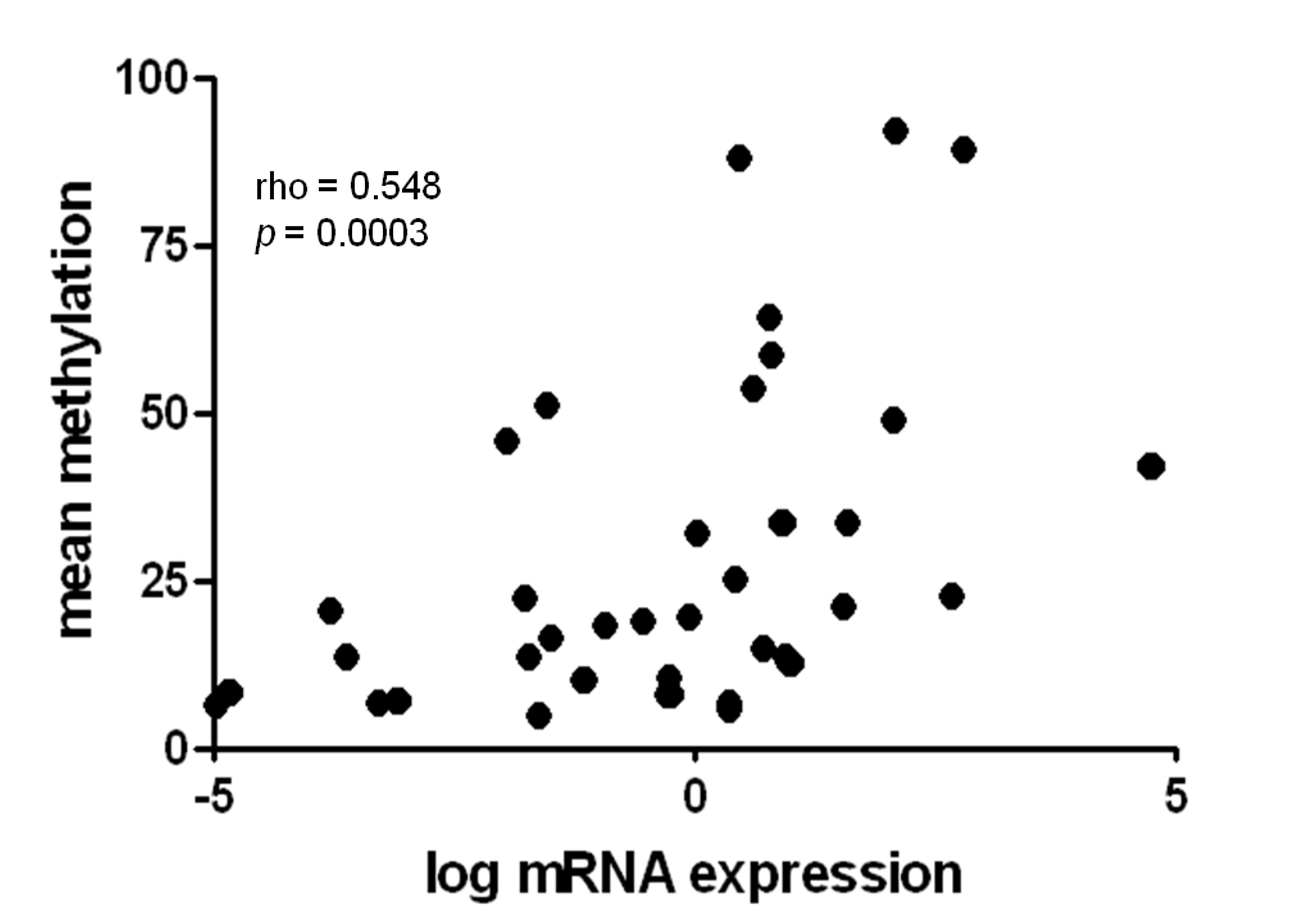

Supplement: Figure S4 — Correlation between UTF1 promoter mean CpG methylation and UTF1 mRNA expression. The mean of CpG methylation was calculated for each sample. Data from qPCR were log transformed before being plotted. Correlation between UTF1 mean CpG methylation and mRNA expression was evaluated by Spearman's rank correlation test, reported p-value was two-sided. (TIF) [file pone.0042704.s004.tif]

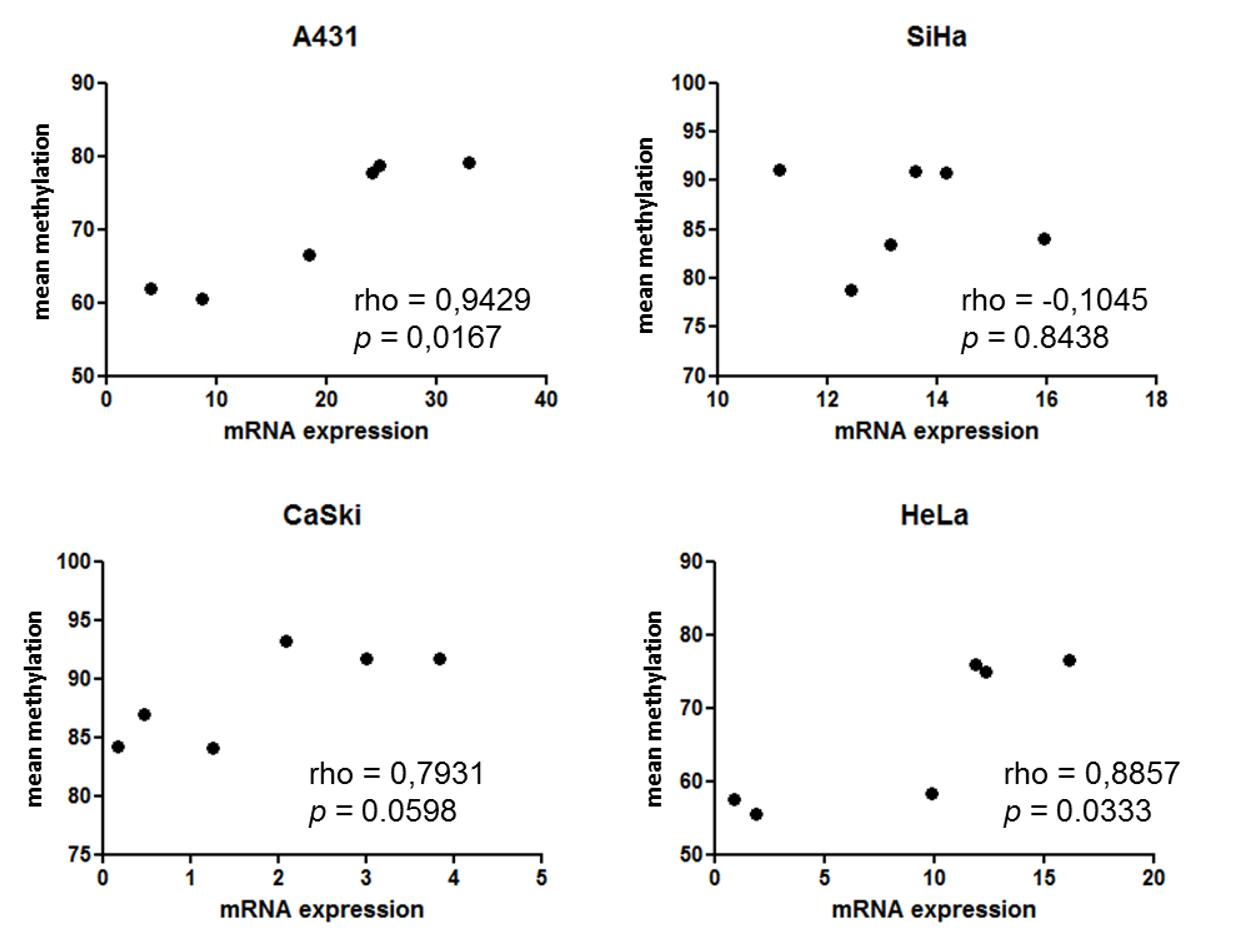

Supplement: Figure S5 — Correlation between UTF1 promoter mean CpG methylation and UTF1 mRNA expression in cancer cell lines treated or not with 5-AZA. The mean of CpG methylation was calculated for each sample. Correlation between UTF1 mean CpG methylation and mRNA expression was evaluated by Spearman's rank correlation test, reported p-value was two-sided. (TIF) [file pone.0042704.s005.tif]

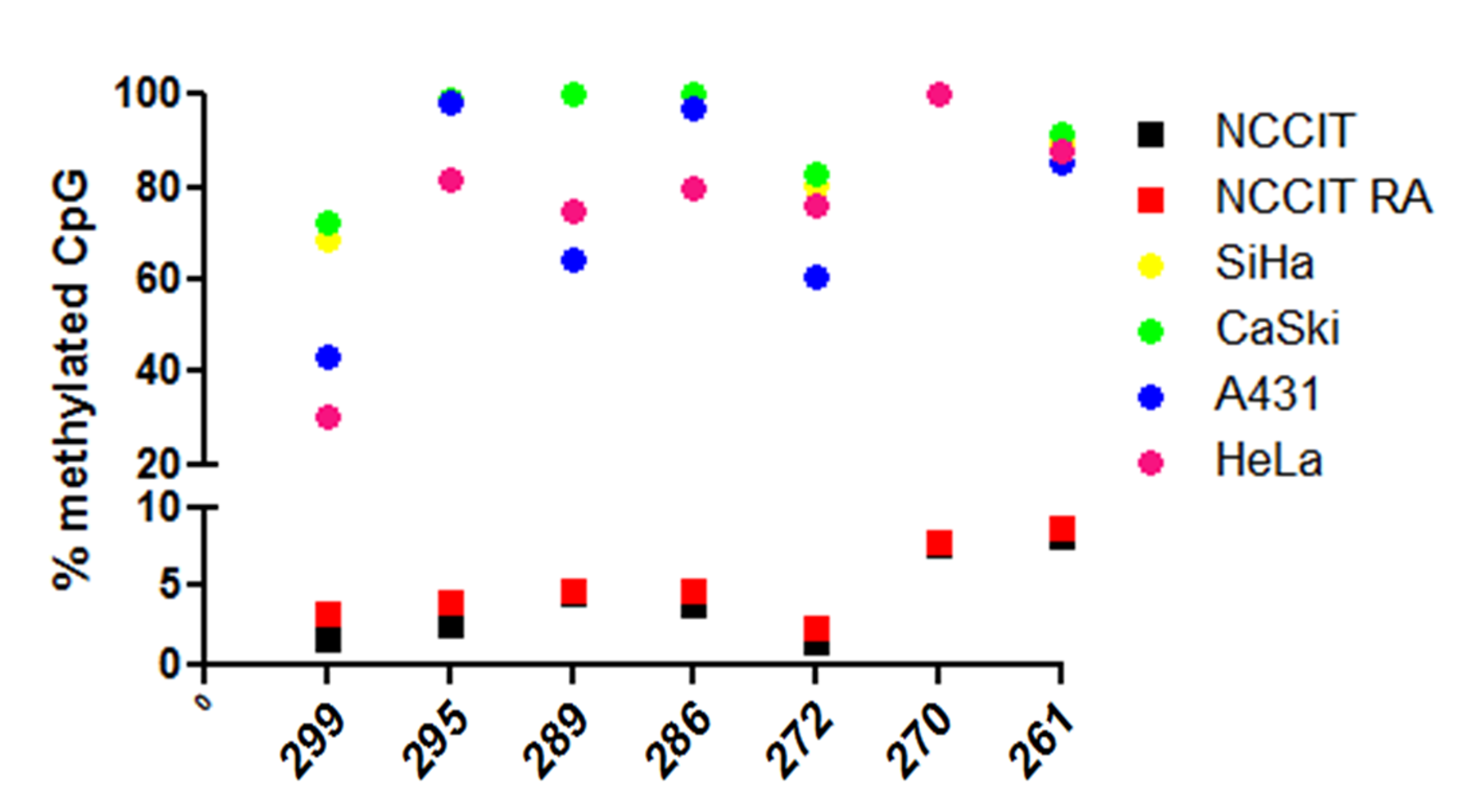

Supplement: Figure S6 — UTF1 promoter methylation analysis by direct bisulfite pyrosequencing in NCCIT and NCCIT differentiated with retinoic acid (NCCIT RA). Position of each CpG is indicated (TSS = +1). For comparison, methylation values for epithelial cancer cell lines used in this work were added. (TIF) [file pone.0042704.s006.tif]
