# Supplementary material for: Aberrant Promoter Methylation and Expression of UTF1 during Cervical Carcinogenesis
Source: PLoS One. 2012 Aug 3;7(8):e42704. doi: 10.1371/journal.pone.0042704 (PMC3411846; doi:10.1371/journal.pone.0042704)
Supplement: Table S1 — The β-values for each CpG of stem cell genes analysed by microarray. (PDF) [file pone.0042704.s007.pdf]

Supplementary Table 1

methylation microarray data performed using Illumina's Infinium Human Methylation27 Beadchip

RefSeqs of Annotated Genomes: Build 36.1

values are comprised beween 0 and 1, with 0 meaning total hypomethylation and 1 meaning total hypermethylation

in yellow are the CpG with Δβ>0,2

SYMBOL	SYNONYM	GENE_ID	TargetID	Ecto1	Ecto2	Ecto3	Ecto4	SCC1	SCC2	SCC3	SCC4	SCC5	Ecto β-mean value	SCC β-mean value	Δβ>0,2
UTF1		GeneID:8433	cg09053680	0,017	0,016	0,015	0,034	0,671	0,693	0,687	0,841	0,634	0,021	0,705	0,685
UTF1		GeneID:8433	cg03755123	0,091	0,075	0,094	0,076	0,628	0,567	0,614	0,745	0,593	0,084	0,629	0,545
MSI1	musashi1	GeneID:4440	cg02883161	0,077	0,062	0,078	0,085	0,221	0,416	0,704	0,251	0,208	0,075	0,360	0,285
DNMT3L	MGC1090;	GeneID:29947	cg27076046	0,470	0,277	0,335	0,292	0,590	0,396	0,732	0,649	0,651	0,343	0,603	0,260
SMO	Gx; SMOH;	GeneID:6608	cg04478795	0,064	0,051	0,064	0,034	0,332	0,214	0,100	0,502	0,398	0,053	0,309	0,256
FGF7	KGF; HBGF-7;	GeneID:2252	cg23504246	0,287	0,136	0,268	0,229	0,442	0,205	0,781	0,480	0,426	0,230	0,467	0,237
SALL4	DRRS; HSAL4; dJ1112F19.1;	GeneID:57167	cg06303238	0,650	0,694	0,626	0,529	0,903	0,647	0,919	0,939	0,898	0,624	0,861	0,237
SOX2	ANOP3; MGC2413;	GeneID:6657	cg15105987	0,169	0,168	0,162	0,201	0,392	0,455	0,392	0,429	0,339	0,175	0,401	0,226
FGF7	KGF; HBGF-7;	GeneID:2252	cg27525902	0,191	0,340	0,275	0,230	0,417	0,254	0,553	0,494	0,382	0,259	0,420	0,161
TDGF1	CR; CRGF; CRIPTO;	GeneID:6997	cg27371741	0,551	0,369	0,488	0,648	0,731	0,520	0,811	0,505	0,756	0,514	0,665	0,151
NES	FLJ21841; Nbla00170;	GeneID:10763	cg21838334	0,104	0,042	0,054	0,032	0,102	0,114	0,566	0,089	0,089	0,058	0,192	0,134
POU5F1	OCT3; OTF3; OTF4; Oct4; MGC22487;	GeneID:5460	cg27388462	0,800	0,815	0,749	0,769	0,887	0,813	0,879	0,876	0,788	0,783	0,849	0,065
EGFR	ERBB; mENA; ERBB1;	GeneID:1956	cg03860890	0,039	0,049	0,023	0,035	0,085	0,066	0,062	0,127	0,134	0,036	0,095	0,058
GNL3	NS; E2IG3; C77032; MGC800;	GeneID:26354	cg11041457	0,078	0,085	0,102	0,107	0,113	0,157	0,177	0,135	0,102	0,093	0,137	0,044
TDGF1	CR; CRGF; CRIPTO;	GeneID:6997	cg10242476	0,414	0,425	0,678	0,415	0,469	0,557	0,545	0,610	0,439	0,483	0,524	0,041
MSI1	musashi1	GeneID:4440	cg20380069	0,039	0,036	0,022	0,028	0,032	0,201	0,024	0,027	0,044	0,031	0,066	0,035
PROM1	AC133; CD133; PROML1; MSTP061;	GeneID:8842	cg20576510	0,782	0,742	0,756	0,736	0,869	0,734	0,800	0,725	0,813	0,754	0,788	0,034
ITGA6	CD49f;	GeneID:3655	cg20795401	0,147	0,099	0,117	0,089	0,179	0,153	0,141	0,127	0,132	0,113	0,146	0,033
GNL3	NS; E2IG3; C77032; MGC800;	GeneID:26354	cg13174197	0,101	0,071	0,109	0,162	0,130	0,154	0,200	0,132	0,096	0,111	0,142	0,031
EGFR	ERBB; mENA; ERBB1;	GeneID:1956	cg14094960	0,070	0,014	0,016	0,023	0,029	0,186	0,031	0,027	0,024	0,031	0,059	0,029
PROM1	AC133; CD133; PROML1; MSTP061;	GeneID:8842	cg21230392	0,838	0,838	0,823	0,789	0,859	0,870	0,856	0,887	0,780	0,822	0,850	0,028
ALDH1A1	ALDC; ALDH1; PUMB1; ALDH11; RALDH1; ALDH-E1; MGC2318;	GeneID:216	cg16601861	0,146	0,132	0,110	0,149	0,146	0,153	0,231	0,152	0,124	0,134	0,161	0,027
PTGS2	COX2; COX-2; PHS-2; PGG/HS; PGHS-2; hCox-2;	GeneID:5743	cg03160309	0,052	0,049	0,056	0,037	0,044	0,039	0,052	0,173	0,068	0,048	0,075	0,027
ITGA1	VLA1; CD49a;	GeneID:3672	cg22625415	0,042	0,039	0,042	0,054	0,034	0,073	0,066	0,112	0,057	0,044	0,068	0,024
CD44	IN; LHR; MC56; MDU2; MDU3; MIC4; Pgp1; CDW44; MUTCH-I; ECMR-III; MGC10468;	GeneID:960	cg08606356	0,064	0,060	0,044	0,050	0,067	0,057	0,067	0,130	0,059	0,055	0,076	0,021
PTGS2	COX2; COX-2; PHS-2; PGG/HS; PGHS-2; hCox-2;	GeneID:5743	cg19155599	0,151	0,072	0,106	0,134	0,100	0,118	0,137	0,142	0,176	0,116	0,135	0,019
POU5F1	OCT3; OTF3; OTF4; Oct4; MGC22487;	GeneID:5460	cg13083810	0,904	0,858	0,879	0,899	0,893	0,899	0,913	0,951	0,857	0,885	0,903	0,018
MYBL2	BMYB; MGC15600;	GeneID:4605	cg23843505	0,075	0,064	0,057	0,066	0,095	0,035	0,120	0,058	0,097	0,065	0,081	0,015
CKAP4	p63; CLIMP-63; MGC99554;	GeneID:10970	cg25591867	0,040	0,024	0,042	0,029	0,062	0,052	0,034	0,033	0,052	0,034	0,047	0,013
KLF4	EZF; GKLF;	GeneID:9314	cg07309102	0,016	0,012	0,012	0,009	0,009	0,040	0,016	0,037	0,022	0,012	0,025	0,012
CD44	IN; LHR; MC56; MDU2; MDU3; MIC4; Pgp1; CDW44; MUTCH-I; ECMR-III; MGC10468;	GeneID:960	cg20143092	0,090	0,087	0,092	0,055	0,114	0,078	0,090	0,098	0,085	0,081	0,093	0,012
CD44	IN; LHR; MC56; MDU2; MDU3; MIC4; Pgp1; CDW44; MUTCH-I; ECMR-III; MGC10468;	GeneID:960	cg01879488	0,026	0,030	0,038	0,047	0,040	0,031	0,056	0,049	0,051	0,035	0,045	0,010
CTNNB1	CTNNB;	GeneID:1499	cg14663065	0,028	0,026	0,032	0,028	0,027	0,037	0,046	0,023	0,055	0,028	0,037	0,009
CD44	IN; LHR; MC56; MDU2; MDU3; MIC4; Pgp1; CDW44; MUTCH-I; ECMR-III; MGC10468;	GeneID:960	cg17640322	0,022	0,019	0,022	0,016	0,021	0,031	0,024	0,026	0,030	0,020	0,027	0,007
MYB	efg; c-myb; c-myb_CDS;	GeneID:4602	cg11579069	0,039	0,052	0,070	0,045	0,042	0,060	0,042	0,095	0,052	0,051	0,058	0,007
SSSCA1	p27;	GeneID:10534	cg22552669	0,020	0,018	0,020	0,025	0,020	0,023	0,019	0,036	0,038	0,021	0,027	0,006
KLF4	EZF; GKLF;	GeneID:9314	cg03939688	0,024	0,013	0,015	0,025	0,024	0,015	0,015	0,020	0,052	0,019	0,025	0,006
NES	FLJ21841; Nbla00170;	GeneID:10763	cg12356261	0,039	0,043	0,049	0,027	0,046	0,041	0,037	0,038	0,061	0,040	0,044	0,005
SMO	Gx; SMOH;	GeneID:6608	cg15447479	0,044	0,025	0,025	0,030	0,030	0,035	0,034	0,052	0,026	0,031	0,035	0,004
PTGS2	COX2; COX-2; PHS-2; PGG/HS; PGHS-2; hCox-2;	GeneID:5743	cg18335243	0,017	0,022	0,050	0,043	0,038	0,026	0,035	0,037	0,048	0,033	0,037	0,004
CD44	IN; LHR; MC56; MDU2; MDU3; MIC4; Pgp1; CDW44; MUTCH-I; ECMR-III; MGC10468;	GeneID:960	cg08530414	0,038	0,029	0,035	0,024	0,038	0,026	0,030	0,025	0,058	0,032	0,036	0,004
ITGA1	VLA1; CD49a;	GeneID:3672	cg21153758	0,014	0,016	0,011	0,018	0,014	0,017	0,017	0,029	0,016	0,015	0,019	0,004
PTGS2	COX2; COX-2; PHS-2; PGG/HS; PGHS-2; hCox-2;	GeneID:5743	cg13986130	0,045	0,032	0,078	0,054	0,068	0,048	0,077	0,047	0,038	0,052	0,055	0,003
ATXN1	ATX1; SCA1; D6S504E;	GeneID:6310	cg10487127	0,011	0,017	0,012	0,028	0,013	0,015	0,025	0,028	0,014	0,017	0,019	0,002
PTGS2	COX2; COX-2; PHS-2; PGG/HS; PGHS-2; hCox-2;	GeneID:5743	cg15774283	0,018	0,016	0,018	0,023	0,012	0,032	0,015	0,022	0,018	0,019	0,020	0,001
SOX2	ANOP3; MGC2413;	GeneID:6657	cg01340005	0,027	0,021	0,018	0,026	0,014	0,018	0,032	0,025	0,028	0,023	0,024	0,000
ITGA6	CD49f;	GeneID:3655	cg10749011	0,018	0,016	0,013	0,014	0,013	0,019	0,012	0,017	0,017	0,015	0,015	0,000
MYC	c-Myc;	GeneID:4609	cg19972619	0,036	0,023	0,018	0,029	0,030	0,024	0,031	0,017	0,030	0,027	0,026	0,000
MYBL2	BMYB; MGC15600;	GeneID:4605	cg09604203	0,019	0,014	0,016	0,013	0,012	0,017	0,010	0,016	0,017	0,016	0,014	-0,001
CD44	IN; LHR; MC56; MDU2; MDU3; MIC4; Pgp1; CDW44; MUTCH-I; ECMR-III; MGC10468;	GeneID:960	cg18652941	0,027	0,031	0,026	0,025	0,023	0,022	0,023	0,016	0,046	0,027	0,026	-0,002
SSSCA1	p27;	GeneID:10534	cg24977709	0,026	0,028	0,023	0,026	0,023	0,021	0,018	0,045	0,013	0,026	0,024	-0,002
PCGF4	BMI1; RNF51; MGC12685;	GeneID:648	cg13984351	0,018	0,035	0,015	0,018	0,016	0,021	0,020	0,018	0,022	0,021	0,019	-0,002
CKAP4	p63; CLIMP-63; MGC99554;	GeneID:10970	cg09239756	0,024	0,022	0,026	0,031	0,024	0,016	0,016	0,034	0,026	0,026	0,023	-0,003
PCGF4	BMI1; RNF51; MGC12685;	GeneID:648	cg13379871	0,012	0,012	0,012	0,018	0,009	0,013	0,008	0,009	0,011	0,014	0,010	-0,004
ATXN1	ATX1; SCA1; D6S504E;	GeneID:6310	cg04542938	0,019	0,017	0,021	0,016	0,011	0,021	0,014	0,011	0,015	0,018	0,014	-0,004
CD44	IN; LHR; MC56; MDU2; MDU3; MIC4; Pgp1; CDW44; MUTCH-I; ECMR-III; MGC10468;	GeneID:960	cg04125208	0,038	0,026	0,016	0,019	0,022	0,018	0,024	0,010	0,027	0,025	0,020	-0,005
CTNNB1	CTNNB;	GeneID:1499	cg04180460	0,024	0,017	0,021	0,029	0,012	0,010	0,022	0,016	0,025	0,023	0,017	-0,006
PTGS2	COX2; COX-2; PHS-2; PGG/HS; PGHS-2; hCox-2;	GeneID:5743	cg09461185	0,035	0,027	0,032	0,020	0,022	0,024	0,019	0,020	0,024	0,028	0,022	-0,006
MYC	c-Myc;	GeneID:4609	cg27207274	0,036	0,045	0,031	0,047	0,034	0,026	0,028	0,029	0,037	0,040	0,031	-0,009
TP53	p53; LFS1; TRP53;	GeneID:7157	cg11519508	0,031	0,027	0,037	0,033	0,021	0,015	0,019	0,025	0,027	0,032	0,022	-0,010
MYB	efg; c-myb; c-myb_CDS;	GeneID:4602	cg18253802	0,038	0,038	0,036	0,030	0,019	0,023	0,027	0,026	0,028	0,035	0,024	-0,011
NOTCH1	hN1; TAN1;	GeneID:4851	cg16829154	0,038	0,033	0,039	0,035	0,025	0,020	0,023	0,023	0,033	0,036	0,025	-0,012
TP53	p53; LFS1; TRP53;	GeneID:7157	cg22175811	0,042	0,054	0,038	0,054	0,018	0,012	0,031	0,025	0,051	0,047	0,027	-0,020
NOTCH1	hN1; TAN1;	GeneID:4851	cg20042228	0,070	0,081	0,051	0,058	0,057	0,038	0,043	0,023	0,060	0,065	0,044	-0,021
GADD45A	DDIT1; GADD45;	GeneID:1647	cg07000785	0,065	0,083	0,069	0,087	0,042	0,046	0,047	0,060	0,074	0,076	0,054	-0,023
GADD45A	DDIT1; GADD45;	GeneID:1647	cg04718306	0,055	0,047	0,066	0,061	0,023	0,025	0,024	0,046	0,050	0,057	0,034	-0,024
ITGB2	LAD; CD18; MF17; MFI7; LCAMB; LFA-1; MAC-1;	GeneID:3689	cg04790129	0,595	0,622	0,618	0,663	0,540	0,519	0,554	0,628	0,368	0,624	0,522	-0,103
ITGB2	LAD; CD18; MF17; MFI7; LCAMB; LFA-1; MAC-1;	GeneID:3689	cg24167037	0,372	0,538	0,562	0,576	0,319	0,294	0,390	0,418	0,368	0,512	0,358	-0,154
