# Supplementary material for: Aberrant Promoter Methylation and Expression of UTF1 during Cervical Carcinogenesis
Source: PLoS One. 2012 Aug 3;7(8):e42704. doi: 10.1371/journal.pone.0042704 (PMC3411846; doi:10.1371/journal.pone.0042704)
Supplement: Table S2 — Values of UTF1 CpG methylation in DNA from frozen tissue by direct bisulfite pyrosequencing. (PDF) [file pone.0042704.s008.pdf]

Supplementary Table S2

the CpG in red was the one analyzed in microarray

methylation values obtained by direct bisulfite pyrosequencing for CpG in DNA from frozen samples

samples	age	hpv load (pg/ml)	CpG1	CpG2	CpG3	CpG4	CpG5	CpG6	CpG7	CpG8
Ecto 1	38	0	5	10	10	9	17	5	25	6
Ecto 2	57	856,38	5	9	8	8	14	6	19	6
Ecto 3	40	112,79	6	10	9	7	11	5	12	8
Ecto 4	35	0	7	23	14	14	34	22	ND	ND
SCC 1	51	15,9	24	30	29	28	37	22	43	17
SCC 2	30	623,41	52	79	72	74	72	51	92	41
SCC 3	42	1555,56	81	89	96	93	100	73	100	63
SCC 4	61	1295,22	49	67	76	74	82	57	94	45
SCC 5	41	766,47	32	47	45	39	59	37	73	27

Statistical analyses

	mean (SD)									
samples	age	hpv load (pg/ml)	CpG1	CpG2	CpG3	CpG4	CpG5	CpG6	CpG7	CpG8
Ecto	42,5 (9,9)	242,3 (412,9)	5.75 (0.96)	13.00 (6.68)	10.25 (2.63)	9.50 (3.11)	19.00 (10.30)	9.5 (8.35)	18.67 (6.51)	6.00 (0)
SCC	45 (11,6)	851,3 (602,1)	47.60 (22.01) [a]	62.40 (23.93) [b]	63.60 (26.54) [c]	61.60 (27.08) [c]	70.00 (23.76) [b]	48.00 (19.44) [a]	80.40 (23.22) [c]	38.80 (17.33) [a]

Statistical test: Two-way ANOVA followed by *post-hoc* Bonferroni Test

a, $p<0.05$ *vs* Ecto

b, $p<0.01$ *vs* Ecto

c, $p<0.001$ *vs* Ecto
