# Supplementary material for: Aberrant Promoter Methylation and Expression of UTF1 during Cervical Carcinogenesis
Source: PLoS One. 2012 Aug 3;7(8):e42704. doi: 10.1371/journal.pone.0042704 (PMC3411846; doi:10.1371/journal.pone.0042704)
Supplement: Table S3 — Values of UTF1 CpG methylation in DNA from liquid-based cytological samples by direct bisulfite pyrosequencing. (PDF) [file pone.0042704.s009.pdf]

Supplementary table S3

the CpG in red was the one analyzed in microarray

methylation values obtained by direct bisulfite pyrosequencing for CpG in DNA from liquid-based cytological samples

samples	age	hpv load (pg/ml)	CpG1	CpG2	CpG3	CpG4	CpG5	CpG6	CpG7	CpG8
Ecto	57	0	8	16	13	13	18	10	17	9
Ecto	57	0	9	23	16	22	23	18	27	19
Ecto	40	7,3	14	18	18	19	25	8	20	9
Ecto	46	0	7	9	10	6	15	8	17	9
Ecto	21	771,27	13	17	14	13	17	13	18	13
Ecto	46	11,56	3	7	5	5	8	4	10	4
Ecto	43	0	2	11	6	6	9	7	12	5
Ecto	85	0	1	8	3	4	7	5	8	3
Ecto	23	49,77	5	8	7	3	12	7	15	6
Ecto	39	5,92	8	14	13	12	17	10	24	11
Ecto	26	0	16	35	39	36	44	24	38	24
Ecto	47	1,57	9	12	10	10	13	9	15	5
Ecto	21	2041,28	14	21	18	18	24	15	23	14
Ecto	27	1520,05	5	7	8	6	9	5	8	4
Ecto	56	0	4	8	6	6	9	6	9	5
Ecto	66	0	12	12	13	11	22	5	19	14
Ecto	34	43,46	18	49	19	19	40	17	27	13
LSIL	39	4,94	20	23	21	19	30	15	24	11
LSIL	24	0	9	11	11	10	14	8	13	7
LSIL	45	0	5	9	8	7	11	7	11	6
LSIL	53	383,78	4	8	7	5	10	5	11	4
LSIL	31	708,1	4	11	9	7	11	5	12	6
LSIL	23	1900	7	9	10	4	20	5	20	6
LSIL	49	157,67	13	26	20	23	29	19	25	14
LSIL	45	13,2	9	17	12	11	16	14	19	10
LSIL	28	4,91	4	10	10	7	12	6	11	6
LSIL	38	4,46	4	9	7	5	12	4	9	6
HSIL	48	1660,21	9	23	16	20	25	15	28	15
HSIL	35	1117,7	16	32	26	23	32	10	25	17
HSIL	47	0	36	39	37	35	41	12	22	26
HSIL	37	111,87	34	35	47	47	51	25	57	39
HSIL	49	147,75	13	20	17	19	42	13	43	12
HSIL	48	959,74	18	21	31	32	64	11	74	17
HSIL	26	34,03	72	79	100	18	100	14	64	67
HSIL	58	0	25	51	35	49	61	45	63	36
HSIL	32	87,01	72	86	78	85	92	73	100	64
HSIL	59	0	27	65	40	52	71	53	65	36
HSIL	22	108,04	7	23	10	9	14	7	19	7
SCC	35	170,53	87	92	91	91	98	80	100	65
SCC	30	11,87	23	41	28	36	44	26	47	23
SCC	42	12,18	28	55	55	50	59	48	61	35
SCC	40	685,59	82	96	95	97	100	81	100	62
SCC	41	42,79	92	96	99	97	100	79	100	73
SCC	77	29,67	24	79	57	36	86	68	79	40
SCC	35	1734,2	49	55	65	66	72	28	58	37
SCC	51	885,2	50	82	76	72	81	70	83	55
SCC	49	3246	53	67	62	64	68	55	75	47

Statistical analyses

	mean (SD)									
samples	age	hpv load (pg/ml)	CpG1	CpG2	CpG3	CpG4	CpG5	CpG6	CpG7	CpG8
Ecto	43,2 (17,5)	261,9 (607,8)	8,67 (5.09)	16,14 (11.23)	12,77 (8.27)	12,22 (8.53)	18,32 (10.62)	10,06 (5.52)	17,98 (8.00)	9,81 (5.76)
LSIL	37,5 (10,6)	317,7 (603,2)	7,92 (5.11)	13,25 (6.49)	11,41 (5.10)	9,66 (9.30)	16,50 (7.36)	8,81 (5.26)	15,62 (5.98)	7,63 (3.18)
HSIL	41,9 (12,3)	384,2 (579,4)	30,02 (22.77) [a]	43,16 (23.84) [c]	39,77 (27.25) [b]	35,42 (21.84) [b]	53,87 (26.93) [c]	25,34 (21.90)	50,80 (26.66) [c]	30,56 (20.26) [a]
SCC	44,4 (13,9)	757,6 (1099,1)	54,22 (27.30) [c,e]	73,54 (20.08) [c,f]	69,69 (22.90) [c,f]	67,64 (23.89) [c,f]	78,63 (19.62) [c,e]	59,41 (21.61) [c,f]	78,01 (19.85) [c,e]	48,57 (16.41) [c,d]

Statistical test: Two-way ANOVA followed by *post-hoc* Bonferroni Test

a, $p < 0.05$ *vs* Ecto and LSIL

b, $p < 0.01$ *vs* Ecto and LSIL

c, $p < 0.001$ *vs* Ecto and LSIL

d, $p < 0.05$ *vs* HSIL

e, $p < 0.01$ *vs* HSIL

f, $p < 0.001$ *vs* HSIL
