# Supplementary material for: Aberrant Promoter Methylation and Expression of UTF1 during Cervical Carcinogenesis
Source: PLoS One. 2012 Aug 3;7(8):e42704. doi: 10.1371/journal.pone.0042704 (PMC3411846; doi:10.1371/journal.pone.0042704)
Supplement: Table S4 — Values of UTF1 CpG methylation in DNA from white blood cells by direct bisulfite pyrosequencing. (PDF) [file pone.0042704.s010.pdf]

Supplementary table S4

the CpG in red was the one analyzed in microarray

methylation values obtained by direct bisulfite pyrosequencing for CpG in DNA from WBC

samples	CpG1	CpG2	CpG3	CpG4	CpG5	CpG6	CpG7
WBC1	4	8	7	7	4	11	11
WBC2	4	9	6	6	3	8	10
WBC3	5	7	6	6	5	10	11
WBC4	6	8	8	6	6	9	10
WBC5	4	11	6	6	4	8	10
WBC6	5	9	8	6	5	10	10
WBC7	4	8	5	5	3	7	8
WBC8	4	7	6	5	4	9	10
WBC9	3	7	6	5	4	9	10
WBC10	4	9	6	5	3	9	8
